# Entangled Schrödinger Bridge Matching

**Published:** 2025-11-10

**Authors:** Sophia Tang, Yinuo Zhang, Pranam Chatterjee

**Affiliations:** 1 Department of Computer and Information Science, University of Pennsylvania; 2 Center of Computational Biology, Duke-NUS Medical School; 3 Department of Bioengineering, University of Pennsylvania

## Abstract

Simulating trajectories of multi-particle systems on complex energy landscapes is a central task in molecular dynamics (MD) and drug discovery, but remains challenging at scale due to computationally expensive and long simulations. Previous approaches leverage techniques such as flow or Schrödinger bridge matching to implicitly learn joint trajectories through data snapshots. However, many systems, including biomolecular systems and heterogeneous cell populations, undergo dynamic interactions that evolve over their trajectory and cannot be captured through static snapshots. To close this gap, we introduce **Entangled Schrödinger Bridge Matching (EntangledSBM)**, a framework that learns the first- and second-order stochastic dynamics of interacting, multi-particle systems where the direction and magnitude of each particle’s path depend dynamically on the paths of the other particles. We define the Entangled Schrödinger Bridge (EntangledSB) problem as solving a coupled system of bias forces that *entangle* particle velocities. We show that our framework accurately simulates heterogeneous cell populations under perturbations and rare transitions in high-dimensional biomolecular systems.

## 1 Introduction

Schrödinger bridge matching (SBM) has enabled significant progress in problems ranging from molecular dynamics (MD) simulation (Holdijk et al., 2023) to cell state modeling (Tong et al., 2024) by effectively learning dynamic trajectories between initial and target distributions. Diverse SBM frameworks have been developed for learning trajectories with minimized state-cost (Liu et al., 2023) to branching trajectories that map from a single initial distribution (Tang et al., 2025a). Most of these frameworks assume that the particle acts independently or undergoes interactions that can be implicitly captured through training on static snapshots of the system. Existing approaches for modeling interacting particle systems rely on the mean-field assumption, where all particles are exchangeable and interact only through averaged effects (Liu et al., 2022; Zhang et al., 2025d). While this assumption may hold for homogeneous particle dynamics, it fails to describe the heterogeneous dynamics observed in more complex domains, such as conformationally dynamic proteins (Vincoff et al., 2025; Chen et al., 2023), heterogeneous cell populations (Smela et al., 2023b), or interacting token sequences (Geshkovski et al., 2023). In these settings, the motion of each particle depends not only on its own position but also on the evolving configuration of surrounding particles.

To accurately model such dependencies, the joint evolution of an n-particle system must be described by a coupled probability distribution that transports the initial distribution $\pi_{0}\left(X_{0}\right)$ to the target distribution $\pi_{ℬ}\left(X_{T}\right)$ in the phase space of both positions and velocities $X_{t}=\left(R_{t},V_{t}\right)\in 𝒳$. However, modeling these interactions requires learning to simulate second-order dynamics, where interactions between velocity fields evolve over time, which remains largely unexplored. To address this gap, we introduce **Entangled Schrödinger Bridge Matching (EntangledSBM)**, a novel framework that learns interacting second-order dynamics of n-particle systems, capturing dependencies on both the static position and dynamic velocities of the system at each time step.

### Contributions

Our contributions can be summarized as follows: **(1)** We formulate the Entangled Schrödinger Bridge (EntangledSB) problem, which considers the optimal path between distributions following second-order Langevin dynamics with an entangled bias force (Sec 3). **(2)** We introduce **EntangledSBM**, a novel parameterization of the bias force that can be conditioned, *at inference*-*time*, on a target distribution or terminal state, enabling generalizable sampling of diverse target distributions (Sec 4). **(3)** We evaluate EntangledSBM on mapping cell cluster dynamics under drug perturbations (Sec 5.1) and transition path sampling of high-dimensional molecular systems (Sec 5.2).

### Related Works

We provide a comprehensive discussion on related works in App A.

## 2 Preliminaries

### Langevin Dynamics

A time-evolving n-particle molecular system can be represented as $X_{t}=(R_{t},V_{t})$, where $r_{t}^{i}\in R^{d}$ denote the coordinates and $v_{t}^{i}\in R^{d}$ are the velocities of each particle i. The evolution of the positions and velocities of the system given a potential energy function U:𝒳→R can be modelled with *Langevin dynamics*, which effectively captures the motion of particles under **conservative forces** between particles and **stochastic collisions** with the surrounding environment (Bussi & Parrinello, 2007; Yang et al., 2006) using a pair of stochastic differential equations (SDEs) defined as

(1) $$dr_{t}^{i}=v_{t}^{i}dt,\;dv_{t}^{i}=\frac{-\nabla_{r_{t}^{i}}U\left(R_{t}\right)}{m_{i}}dt-\gamma v_{t}^{i}dt+\sqrt{\frac{2\gamma k_{B}\tau }{m_{i}}}dW_{t}^{i}$$

where $m_{i}$ is the mass of each particle, γ is the friction coefficient, τ is the temperature, and $dW_{t}$ is standard Brownian motion. In molecular dynamics (MD) simulations of biomolecules, the particles undergo *underdamped* Langevin dynamics with small γ, where *inertia* is not negligible.

Many biological systems, including cell clusters for cell-state trajectory simulation, can be modeled with *overdamped* Langevin dynamics, where inertia is negligible but the system still undergoes external forces that define its motion. This can be represented with the first-order SDE given by

(2) $$dr_{t}^{i}=\frac{-\nabla_{r_{t}^{i}}U\left(R_{t}\right)}{\gamma }dt+\sqrt{\frac{2k_{B}\tau }{\gamma }}dW_{t}^{i}$$

### Schrödinger Bridge Matching

Tasks such as simulating cell state trajectories and transition path sampling aim to simulate the Langevin dynamics from an initial distribution to a desired target state or distribution. Given an initial distribution $\pi_{𝒜}$ and a target distribution $\pi_{ℬ}$, we define the distribution of paths $X_{0:T}≔{\left(X_{t}\right)}_{t\in [0,T]}$ satisfying the endpoint constraints $R_{0}\sim \pi_{𝒜}$ and $R_{T}\sim \pi_{ℬ}$ as the *optimal bridge distribution* $P^{⋆}\left(X_{0:T}\right)$ defined as

(3) $$P^{⋆}\left(X_{0:T}\right)=\frac{1}{Z}P^{0}\left(X_{0:T}\right)\pi_{ℬ}\left(R_{T}\right),\;Z=E_{X_{0:T}\sim P^{0}}\left[\pi_{ℬ}\left(R_{T}\right)\right]$$

where $P^{0}\left(X_{0:T}\right)$ is the base path distribution generated from the SDEs (1) or (2) and Z is the normalizing constant. Schrödinger Bridge Matching (SBM) aims to parameterize a **control** or **bias force** $b_{\theta }$ that tilts the path distribution $P^{b_{\theta }}\left(X_{0:T}\right)$ minimizes the KL-divergence from the bridge path distribution $P^{⋆}$ given by

(4) $$b^{⋆}=\text{arg}\;\underset{b_{\theta }}{\text{min}}\;D_{\text{KL}}\left(P^{b_{\theta }}\|P^{⋆}\right)\;\text{s.t.}\;\left\{\begin{array}{l} dr_{t}^{i}=v_{t}^{i}dt\\ dv_{t}^{i}=\frac{-\nabla_{r_{t}^{i}}U\left(R_{t}\right)+b_{\theta }\left(R_{t}\right)}{m_{i}}dt-\gamma v_{t}^{i}dt+\sqrt{\frac{2\gamma k_{B}\tau }{m_{i}}}dW_{t}^{i} \end{array}\right.$$

In the case of transition path sampling (TPS) where there is a single initial state $R_{𝒜}$ and target state $R_{ℬ}$, we define the target distribution as the relaxed indicator function $\pi_{ℬ}\left(R_{T}\right)=1_{ℬ}\left(R_{T}\right)$ centered around $R_{ℬ}$.

## 3 Learning Interacting Multi-Particle Dynamics

The **key challenge** with simulating the dynamics of multi-particle systems lies in the question: *how can we simulate dynamic trajectories from static snapshot data?* The emergence of flow and Schrödinger bridge matching frameworks have effectively approached this problem by defining a **parameterized velocity field** that learns feasible trajectories from data snapshots. However, current strategies remain limited in their ability to model *interacting* multi-particle systems where the velocities carry inherent dependencies that cannot be captured via static snapshots of the system. Prior attempts to capture interactions between particles rely on the *mean*-*field assumption*, where each particle acts as an average of its surrounding particles, which **does not hold for heterogeneous biomolecular systems**.

To address this challenge, we propose a framework that introduces an additional degree of freedom through a **bias force** that implicitly captures the dependencies between *both* the static positions and dynamic velocities of each particle in the system to control the joint trajectories. We leverage a Transformer architecture and treat each particle as an individual token with features that attend to the features of all other tokens. Crucially, our approach requires no handcrafted features, making it scalable to high-complexity systems.

In Sec 3.1, we formalize this problem as the **Entangled Schrödinger Bridge (EntangledSB)** problem, which aims to determine the optimal set of trajectories to a target state while capturing the dynamic interactions between particles. We then provide a tractable approach to solve the EntangledSB problem with stochastic optimal control (SOC) theory in Sec 3.2. We illustrate the high-level framework of learning **entangled** bias forces in Alg 1 and detail our specific implementation and parameterization in Sec 4.

### 3.1 Entangled Schrödinger Bridge Problem

Here, we formalize the **Entangled Schrödinger Bridge (EntangledSB) problem**, which aims to find a set of optimal bias forces for each particle in the system that depends *dynamically* on the positions and velocities across the entire multi-particle system to guide the Schrödinger bridge trajectory to a target distribution. Specifically, we consider an n-particle system $X_{t}=\left(R_{t},V_{t}\right)$ with $R_{t}={\left\{r_{t}^{i}\in R^{d}\right\}}_{i=1}^{n}$ and $V_{t}={\left\{v_{t}^{i}\in R^{d}\right\}}_{i=1}^{n}$, where each particle evolves over the time horizon t∈[0,T] via the **bias-controlled** SDE given by

(5) $$dr_{t}^{i}=\frac{1}{\gamma }\left(-\nabla_{r_{t}^{i}}U\left(R_{t}\right)+b^{i}\left(R_{t},V_{t}\right)\right)dt+\sqrt{\frac{2k_{B}\tau }{\gamma }}dW_{t}^{i}$$

The velocities follow the potential energy landscape defined over the joint coordinates of the system $U\left(R_{t}\right)$ with an *entangled* bias force $b^{i}\left(R_{t},V_{t}\right)$ that depends dynamically on the position and velocity of *all* particles in the system. We can further extend this framework to **second-order** Langevin dynamics via the pair of SDEs

(6) $$dr_{t}^{i}=v_{t}^{i}dt,\;dv_{t}^{i}=\frac{-\nabla_{r_{t}^{i}}U\left(R_{t}\right)+b^{i}\left(R_{t},V_{t}\right)}{m_{i}}dt-\gamma v_{t}^{i}dt+\sqrt{\frac{2\gamma k_{B}\tau }{m_{i}}}dW_{t}^{i}$$

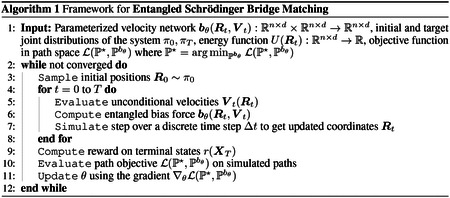


Formally, we seek the optimal $b^{⋆}\left(R_{t},V_{t}\right)≔{\left\{b^{i}\left(R_{t},V_{t}\right)\right\}}_{i=1}^{n}$ that solves the EntangledSB problem defined below.

**Definition 3.1** (Entangled Schrödinger Bridge Problem). *Given an initial distribution $\pi_{𝒜}$*, *a target distribution* $\pi_{ℬ}$, *the EntangledSB problem aims to determine the set of optimal bias forces* $b^{⋆}≔{\left\{b^{i}\left(R_{t},V_{t}\right)\right\}}_{i=1}^{n}$ *for each particle* i *in the system that solves the optimization problem*

(7) $$b^{⋆}\left(R_{t},V_{t}\right)=\text{arg}\;\underset{b_{\theta }}{\text{min}\;}D_{KL}\left(P^{b_{\theta }}\|P^{⋆}\right)\;\text{s.t.}\;\left\{\begin{array}{l} P^{⋆}=\frac{1}{Z}P^{0}\pi_{ℬ}\left(X_{T}\right)\\ P_{0}^{⋆}=\pi_{𝒜}\left(X_{0}\right) \end{array}\right.$$

*where* $P^{0}$ *is the base path measure with joint dynamics that follow the SDEs in* (6).

### 3.2 Solving EntangledSB with Stochastic Optimal Control

To solve the EntangledSB problem, we leverage stochastic optimal control (SOC) theory where we aim to find the set of optimal control drifts $b^{⋆}\left(R_{t},V_{t}\right)$ given a target distribution $\pi_{ℬ}$.

**Proposition 3.1** (Solving EntangledSB with Stochastic Optimal Control). *We can solve the EntangledSB problem with the stochastic optimal control (SOC) objective given by*

(8) $$b^{⋆}=\text{arg}\;\underset{b_{\theta }}{\text{min}\;}E_{X_{0:T}\sim P^{b_{\theta }}}\left[\int_{0}^{T}\frac{1}{2}{\left\|b_{\theta }\left(R_{t},V_{t}\right)\right\|}^{2}dt-r\left(X_{T}\right)\right]\;\text{s.t.}\;(6)$$

*where* $r\left(X_{T}\right)≔\text{log}\;\pi_{ℬ}\left(R_{T}\right)$ *is the terminal reward that measures the log*-*probability under the target distribution*.

The proof is provided in App C.1. We highlight that the SOC problem is optimized over *unconstrained* trajectories that need not map to explicit samples from $\pi_{ℬ}$ but are iteratively refined to generate trajectories with a terminal distribution that matches $\pi_{ℬ}$. In Sec 4.2, we introduce an importanceweighted cross-entropy objective that efficiently solves the SOC problem with theoretical guarantees.

## 4 Entangled Schrödinger Bridge Matching

In this section, we introduce **Entangled Schrödinger Bridge Matching** (EntangledSBM), a novel framework for learning to simulate trajectories of n-particle systems that with a **bias force that dynamically depends on the positions and velocities of the other particles** in the system and can generalize to *unseen* target distributions without further training. Our unique parameterization ensures a non-increasing distance toward the target distribution without sacrificing expressivity with hard constraints (Sec 4.1). We introduce a **weighted cross-entropy** objective that enables efficient *off*-*policy learning* with a replay buffer of simulated trajectories (Sec 4.2).

### 4.1 Parameterizing the Entangled Bias Force

#### Bias Force Parameterization

To ensure that the bias force does not increase the distance from the target distribution $\pi_{ℬ}$, we ensure that the projection of the predicted bias force onto the direction of the gradient of the target distribution is positive for all particles i in the system

(9) $$\left\langle b_{\theta }^{i}\left(R_{t},V_{t}\right),\nabla_{r_{t}^{i}}\;\text{log}\;\pi_{ℬ}\right\rangle \geq 0$$

Let $s_{i}=\nabla_{r_{t}^{i}}\;\text{log}\pi_{ℬ}\in R^{d}$ denote the direction towards the target state. To ensure that the bias force is within the cone surrounding $s_{i}$, we use the following parameterization

(10) $$b_{\theta }^{i}\left(R_{t},V_{t}\right)=\underset{\text{parallel}\;\text{component}}{\underbrace{\alpha_{\theta }^{i}\left(R_{t},V_{t}\right){\overset{ˆ}{s}}_{i}}}+\underset{\text{orthogonal}\;\text{component}}{\underbrace{\left(I-{\overset{ˆ}{s}}_{i}{\overset{ˆ}{s}}_{i}^{⊤}\right)h_{\theta }^{i}\left(R_{t},V_{t}\right)}},\;\text{where}\;{\overset{ˆ}{s}}_{i}=\frac{s_{i}}{\left\|s_{i}\right\|}$$

where $\alpha_{\theta }^{i}\left(R_{t},V_{t}\right)≔\text{softplus}\left(\alpha_{\theta }^{i}\left(R_{t},V_{t}\right)\right)\geq 0$ is a scaling factor for the unit vector ${\overset{ˆ}{s}}_{i}$ and $h_{\theta }^{i}\left(R_{t},V_{t}\right)\in R^{d}$ is the per-atom correction vector that is projected onto the plane orthogonal to ${\overset{ˆ}{s}}_{i}$, both parameterized with neural networks θ. Since the orthogonal component does not affect the dot product with ${\overset{ˆ}{s}}_{i}$, the non-negativity constraint $\left\langle b_{\theta }^{i}\left(R_{t},V_{t}\right),\nabla_{r_{t}^{i}}\;\text{log}\;\pi_{ℬ}\right\rangle \geq 0$ in (9) is guaranteed by the first term. Intuitively, the orthogonal component enables the bias force to have greater flexibility in moving sideways (e.g., to avoid infeasible regions or add rotational/collective effects) while ensuring that the distance from some target state remains non-increasing.

**Proposition 4.1** (Non-Increasing Distance from Target Distribution). *For small enough* Δt, *the distance from some target state* $R_{ℬ}\in \pi_{ℬ}$ *state after an update with the bias force* $b_{\theta }^{i}\left(R_{t},V_{t}\right)$ *defined in* (10) *is non-increasing, such that*

(11) $$\exists R_{ℬ}\in \pi_{ℬ}\;\text{s.t.}\;\left\|R_{t+\Delta t}-R_{ℬ}\right\|\leq \left\|R_{t}-R_{ℬ}\right\|$$

*where* $R_{t+\Delta t}=R_{t}+\left(b_{\theta }^{i}\left(R_{t},V_{t}\right)/m_{i}\right)\Delta t$.

The proof is provided in App C.2. In contrast to previous works that constrain the bias force to point strictly in the direction of a fixed target position, our approach allows greater flexibility in the direction of the orthogonal component.

#### Model Architecture

To integrate dependencies on the positions and velocities across n particles, we leverage a Transformer-based architecture where each particle has input features $\text{Cat}\left(r_{t}^{i},v_{t}^{i}\right)\in R^{2d}$ and the full system features are $\text{Cat}\left(R_{t},V_{t}\right)\in R^{n\times 2d}$. We further input the direction $R_{ℬ}-R_{t}$ and distance $\left\|R_{ℬ}-R_{t}\right\|$ from the target distribution, which enables generalization to unseen target distributions at inference by learning the dependence of the bias force on the target direction. The Transformer encoder enables efficient and expressive propagation of feature information across all pairs of particles in the system to generate context-aware embeddings for bias force parameterization. For MD systems, we ensure invariance of the coordinate frame using the Kabsch algorithm (Kabsch, 1976), which aligns the position $R_{t}$ with the target position $R_{ℬ}$ before input into the model. Further details are provided in App D and E.

### 4.2 Off-Policy Learning with Weighted Cross-Entropy

#### Log-Variance Objective

To train the bias force to match the optimal $b^{⋆}$, we can adapt the *log*-*variance* (LV) divergence (Seong et al., 2025; Nüsken & Richter, 2021) defined as

(12) $${ℒ}_{\text{LV}}\left(P^{⋆},P^{b_{\theta }}\right)={\text{Var}}_{P^{v}}\left[\text{log}\frac{\text{d}P^{⋆}}{\text{d}P^{b_{\theta }}}\right]=E_{P^{v}}\left[{\left(\text{log}\frac{\text{d}P^{⋆}}{\text{d}P^{b_{\theta }}}-E_{P^{v}}\left[\text{log}\frac{\text{d}P^{⋆}}{\text{d}P^{b_{\theta }}}\right]\right)}^{2}\right]$$

where v is an arbitrary control that enables *off*-*policy learning* from trajectories that need not be generated by the current bias force $b_{\theta }$. While the optimal solution to the LV objective is exactly the optimal bias force $b^{⋆}$, the non-convex nature of the LV objective with respect to the path measure $P^{b_{\theta }}$ inhibits convergence. We provide more details in App B.

#### Cross-Entropy Objective

To achieve a more theoretically-favorable optimization problem, we propose a cross-entropy objective that is *convex* with respect to the biased path measure $P^{b_{\theta }}$ given by

(13) $${ℒ}_{\text{CE}}\left(P^{⋆},P^{b_{\theta }}\right)=E_{P^{⋆}}\left[-\text{log}\;P^{b_{\theta }}\right]≔D_{\text{KL}}\left(P^{⋆}\|P^{b_{\theta }}\right)=E_{P^{⋆}}\left[\text{log}\frac{\text{d}P^{⋆}}{\text{d}P^{b_{\theta }}}\right]=E_{P^{v}}\left[\frac{\text{d}P^{⋆}}{\text{d}P^{v}}\text{log}\frac{\text{d}P^{⋆}}{\text{d}P^{b_{\theta }}}\right]$$

where $P^{⋆}$ is the target bridge measure and $P^{v}$ is the path measure generated with an arbitrary control v. To avoid taking the expectation with respect to the unknown path measure $P^{⋆}$, we define an importance weight $w^{⋆}\left(X_{0:T}\right)≔\frac{\text{d}P^{⋆}}{\text{d}P^{v}}\left(X_{0:T}\right)$ independent of $b_{\theta }$ that enables optimization using trajectories generated from an arbitrary control v. We note that similar CE objectives have been adopted in earlier work for different applications (Kappen & Ruiz, 2016; Zhu et al., 2025; Tang et al., 2025b), which we discuss in App A.

**Proposition 4.2** (Convexity and Uniqueness of Cross-Entropy Objective). *The cross*-*entropy objective* ${ℒ}_{CE}$ *is convex in* $P^{b_{\theta }}$ *and there exists a unique minimizer* $b^{⋆}$ *that is the solution to the EntangledSOC problem in Proposition 3.1*.

The proof is given in App C.3. To amortize the cost of simulation and reinforce high-reward trajectories, we define the arbitrary control as the biased path measure from the previous iteration $v≔\bar{b}=\text{stopgrad}\left(b_{\theta }\right)$, which allows us to reuse the trajectories over multiple training steps by maintaining a replay buffer ℛ that contains the trajectories $X_{0:T}$ and their importance weights.

**Proposition 4.3** (Equivalence of Variational and Path Integral Objectives). *The cross*-*entropy objective can be expressed in path*-*integral form as*

(14) $${ℒ}_{CE}(\theta )=E_{P^{v}}\left[w^{⋆}\left(X_{0:T}\right){ℱ}_{b_{\theta },v}\left(X_{0:T}\right)\right]$$

(15) $$\{\begin{array}{l} w^{⋆}\left(X_{0:T}\right)=\frac{\text{d}{\mathbb{P}}^{⋆}}{\text{d}{\mathbb{P}}^{v}}\left(X_{0:T}\right)=\frac{e^{r\left(X_{T}\right)}}{Z}\frac{\text{d}{\mathbb{P}}^{0}}{\text{d}{\mathbb{P}}^{v}}\left(X_{0:T}\right)\\ F_{b_{\theta },v}\left(X_{0:T}\right)=\frac{1}{2}\int_{T}^{0}{\left\|b_{\theta }\left(R_{t},V_{t}\right)\right\|}^{2}-\int_{T}^{0}\left(b_{\theta }^{⊤}v\right)\left(R_{t},V_{t}\right)dt-\int_{T}^{0}b_{\theta }{\left(R_{t},V_{t}\right)}^{⊤}dW_{t} \end{array}$$

*where we define the reference measure* $v=\bar{b}≔\text{stopgrad}\left(b_{\theta }\right)$ *is the off*-*policy control drift from the previous iteration*.

The proof is given in App C.4. Since the normalizing constant Z is intractable in practice, we compute the importance weight as $w^{⋆}\left(X_{0:T}\right)≔{\text{softmax}}_{ℬ}\left(r\left(X_{T}\right)+\text{log}\;p^{0}\left(X_{0:T}\right)-\text{log}\;p^{\overset{‾}{b}}\left(X_{0:T}\right)\right)$, which is a batch estimate of $\frac{{\text{d}p}^{⋆}}{{\text{d}p}^{\overset{‾}{b}}}$. When a batch contains only a single trajectory (i.e., when the system is large), we additionally store the value of $\text{log}\;r\left(X_{T}\right)$ sample from the importance weighted distribution over the replay buffer as $X_{0:T}\sim \text{Cat}\left({\text{softmax}}_{ℛ}\left(w^{⋆}\left(X_{0:T}\right)\right)\right)$.

#### Discrete Time Objective

Since we want to aim to train on off-policy trajectories from previous iterations to dynamically update the learned bias force given the velocities of the remaining particles, we require storing the simulated trajectories in a discretized form. To compute the term ${ℱ}_{b_{\theta },\bar{b}}$ in the CE loss, we discretize it as

(16) $${\hat{ℱ}}_{b_{\theta },\bar{b}}\left(X_{0:K}\right)=\frac{1}{2}\sum_{k=0}^{K-1}{\left\|b_{\theta }\left(X_{k}\right)\right\|}^{2}\Delta t-\sum_{k=0}^{K-1}\left(b_{\theta }\cdot \bar{b}\right)\left(X_{k}\right)\Delta t-\sum_{k=0}^{K-1}b_{\theta }\left(X_{k}\right)\cdot \Delta W_{k}$$

where $\Delta W_{k}=\Sigma^{-1}\left[R_{k+1}-R_{k}-\left(f\left(X_{k}\right)+\Sigma \bar{b}\left(R_{k},V_{k}\right)\right)\Delta t\right]$. Now, we can establish a simplified version of the cross-entropy objective in Prop 4.3.

**Proposition 4.4** (Discretized Cross-Entropy). *Given the discretized* ${\hat{ℱ}}_{b_{\theta },\bar{b}}\left(X_{0:K}\right)$ *in* (16), *we can derive a simplified loss function as*

(17) $${\hat{ℒ}}_{CE}(\theta )=E_{X_{0:K}\sim P^{\overset{‾}{b}}}[\underset{w^{⋆}\left(X_{0:K}\right)}{\underbrace{\frac{\text{d}P^{⋆}}{\text{d}P^{\overset{‾}{b}}}\left(X_{0:K}\right)}}\;\text{log}\left(\frac{p^{0}\left(X_{0:K}\right)\;\text{exp}\left(r\left(X_{K}\right)\right)}{p^{b}\left(X_{0:K}\right)}\right)]$$

*where* $w^{⋆}\left(X_{0:K}\right)$ *is the importance weight of the discrete time trajectory* $X_{0:K}$.

The proof is provided in App C.5. This allows us to train by sampling trajectories, tracking their log-probabilities under the biased and base path measure, computing the terminal reward $r\left(X_{K}\right)$, storing the values in the replay buffer ℛ, and reusing the trajectories over $N_{\text{epochs}}$ training iterations.

## 5 Experiments

We evaluate **EntangledSBM** on several trajectory simulation tasks involving n-particle systems with interacting dynamics, including simulating cell cluster dynamics under drug perturbation (Sec 5.1) and transition path sampling (TPS) for molecular dynamics (MD) simulation of proteins (Sec 5.2).

### 5.1 Simulating Interacting Cell Dynamics Under Perturbation

Populations of cells undergo dynamic signaling interactions that cause shifts in cell state. Under a drug perturbation, these interactions determine the perturbed state of the cell population, which is of significant importance in drug discovery and screening. In this experiment, we use **EntangledSBM** to parameterize an *entangled* bias force that learns **dynamic interactions between cells**, guiding the trajectories of a batch of cells to the perturbed state. We demonstrate that EntangledSBM can not only accurately reconstruct the perturbed cell states within the training distribution following trajectories on the data manifold, but can also generate paths to *unseen* target distributions that diverge from the training distribution, demonstrating its potential to **scale to diverse perturbations and cell types with sparse data**.

#### Setup and Baselines

To model cell state trajectories with high resolution, we evaluate two perturbations (Clonidine and Trametinib at 5μL) from the Tahoe-100M dataset (Zhang et al., 2025a), each containing data for a total of 60K genes. We select the top 2000 highly variable genes (HVGs) and perform principal component analysis (PCA), to maximally capture the variance in the data via the top principal components (38% in the top-50 PCs). To evaluate the ability of EntangledSBM to simulate trajectories to *unseen* target cell clusters that diverge in its distribution from the training target distribution, we further cluster the perturbed cell data to construct multiple disjoint perturbed cell populations. For Clonidine, we generated two clusters, and for Trametinib, we generated three clusters (Figure 5). For each experiment, we trained on a single cluster, and the remaining clusters were left for evaluation.

We trained the bias force given batches of cell clusters with n=16 cells, where the initial cluster is sampled from the unperturbed cell data $\pi_{𝒜}$, and the target cluster is sampled from one of the perturbed cell populations $\pi_{ℬ}$. Each batch of cells $X_{t}=\left(R_{t},V_{t}\right)$ has positions $R_{t}\in R^{n\times d}$ and velocities $V_{t}\in R^{n\times d}$, where d is the dimension of the principal components (PCs) that we simulate. We define the energy landscape such that regions of high data density have low energy and the base dynamics $P^{0}$ follow the gradient toward high data density as detailed in App D.1. We evaluate the reconstruction accuracy of the perturbed distribution at t=T using the maximum mean discrepancy (**RBF-MMD**) for all d PCs and Wasserstein distances (${𝒲}_{1}$ and ${𝒲}_{2}$) of the top two PCs between ground truth and reconstructed clusters after 100 simulation steps with details in D.2. To demonstrate the significance of entangled velocity conditioning on performance, we trained our model with only the positions as input $b_{\theta }\left(R_{t}\right)$. We additionally compare our cross-entropy objective ${ℒ}_{\text{CE}}$ from Sec 4.2 with the log-variance divergence ${ℒ}_{\text{LV}}$ in App F. Additional experiment details are provided in App D.

#### Clonidine Perturbation Results

**Figure 2:**
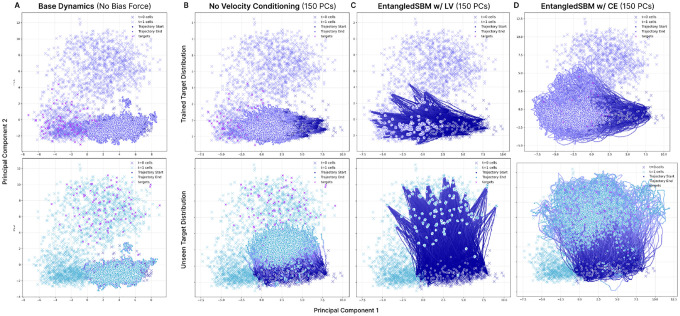
Simulated cell cluster dynamics with EntangledSBM under Clonidine perturbation with 150 PCs. Nearest neighbour cell clusters with n=16 cells are simulated over 100 time steps to perturbed cells seen during training (**Top**) and an unseen perturbed population (**Bottom**). The gradient indicates the evolution of timesteps from the initial time t=0 (navy) to the final time t=T (purple or turquoise). **(A)** Trajectories under base dynamics with no bias force. **(B)** Trajectories simulated with base and bias forces with **(B)** no velocity conditioning, **(C)** log-variance (LV) objective, and **(D)** cross-entropy (CE) objective.

We demonstrate that EntangledSBM accurately guides the base dynamics, which largely remain in the initial data-dense state, to the target perturbed state across increasing PC dimensions d={50,100,150} and reconstructs the target distribution (Fig. 2; Table 1). Notably, we show that our parameterization of the bias force enables generalization to **unseen perturbed populations** that *diverge* from the training distribution by learning dependencies on the target state (Fig. 2). Comparing the reconstruction metrics of the target perturbed cell states with and without velocity conditioning, we confirm our hypothesis that introducing dependence on the dynamic velocities across a cell cluster enables more accurate reconstruction of the target distribution compared to when the bias term is trained with only positional dependence (Table 1). Furthermore, we observe that the LV objective ${ℒ}_{\text{LV}}$ generates nearly straight abrupt trajectories to the target state while the CE objective ${ℒ}_{\text{CE}}$ generates smooth paths along the data manifold (Table 6; Fig. 7), demonstrating its superiority as an **unconstrained objective** for learning controls for optimizing path distributions as further discussed in App F.

#### Trametinib Perturbation Results

**Figure 3:**
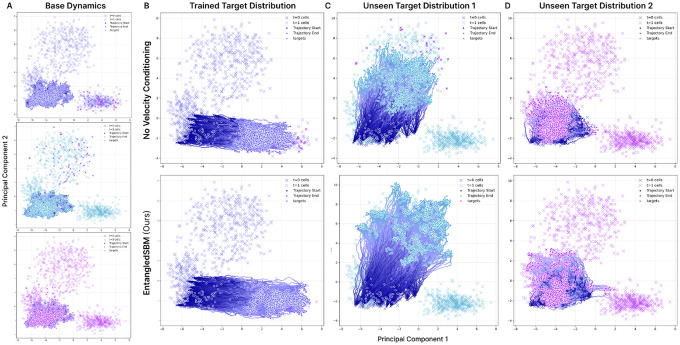
Comparison of EntangledSBM with and without velocity conditioning for cell cluster simulation under Trametinib perturbation. 50 PCs are simulated with the learned bias force trained with the CE objective without velocity conditioning (**Top**) and with velocity conditioning (**Bottom**) to **(B)** the perturbed population used for training and (**C, D**) the two unseen target populations.

We further evaluate our method to predict trajectories of cell clusters under perturbation with Trametinib, which induces three distinct perturbed cell distributions (Figure 5B). Despite training on only one of the perturbed distributions, we demonstrate that EntangledSBM trained with ${ℒ}_{\text{CE}}$ is capable of accurately reconstructing the remaining cell distributions *without* additional training, demonstrating significantly improved performance compared to the bias force trained without velocity conditioning (Table 1; Fig. 3). Furthermore, we observe the same phenomena observed for Clonidine when training with the LV objective, which results in trajectories that fail to capture the intermediate cell dynamics (Table 7; Fig. 8).

### 5.2 Transition Path Sampling of Protein Folding Dynamics

Simulating rare transitions that occur over long timescales and between metastable states on molecular dynamics (MD) landscapes remains a significant challenge due to the high feature dimensionality of biomolecules. In this experiment, we aim to simulate feasible transition paths across high-energy barriers at an *all*-*atom* resolution, a task that is challenging for both traditional MD and ML-based methods. We demonstrate that EntangledSBM generates feasible transition paths with higher target accuracy against a range of baselines.

#### Setup and Baselines

We follow the setup in Seong et al. (2025) and simulate the position and velocity using OpenMM (Eastman et al., 2017) with an annealed temperature schedule. To evaluate the performance of EntangledSBM, we consider the RMSD of the Kabsch-aligned coordinates averaged across 64 paths (**RMSD**; Å) (Kabsch, 1976), percentage of simulated trajectories that hit the target state (**THP**; %), and the highest energy transition state along the biased trajectories averaged across the trajectories that hit the target (**ETS**; kJ/mol). For baselines, we compare against unbiased MD (**UMD**) with temperatures of 3600K for alanine dipeptide and 1200K for the fast-folding proteins, steered MD (**SMD**; Schlitter et al. (1994); Izrailev et al. (1999)) with temperatures of 10K and 20K, path integral path sampling (**PIPS**; Holdijk et al. (2023)), transition path sampling with diffusion path samplers (**TPS-DPS**; Seong et al. (2025)) with the highest performing *scaled* parameterization. Additional experiment details are provided in App E.

#### Alanine Dipeptide

**Figure 4:**
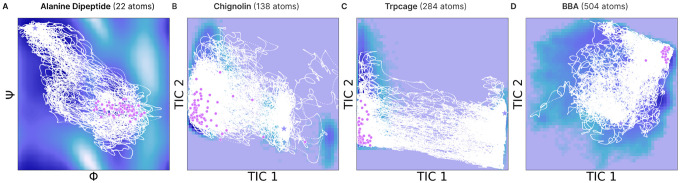
Transition paths generated with EntangledSBM. The energy landscape is colored from dark navy (low potential energy) to light purple (high potential energy) and plotted with the backbone dihedral angles (ϕ,ψ) for Alanine Dipeptide and the top two TICA components for the fast-folding proteins. The starting state $R_{0}$ and target states $R_{ℬ}$ are indicated with purple stars, and the final states of the simulated trajectories $R_{T}$ are indicated with pink circles.

First, we consider Alanine Dipeptide with two alanine residues and 22 atoms. We aim to simulate trajectories to the target state $\pi_{ℬ}=\left\{R|\left\|\xi (R)-\xi \left(R_{ℬ}\right)\right\|<0.75\right\}$ defined by the backbone dihedral angles ξ(R)=(ϕ,ψ). We show that EntangledSBM generates feasible trajectories through both saddle points, representing the two reaction channels (Figure 4A), achieving superior target hit potential (THP) and lower root mean squared error (RMSD) from the target state than all baselines (Table 2).

#### Fast-Folding Proteins

We evaluate EntangledSBM on the more challenging task of modeling the all-atom transition paths of four fast-folding proteins, including **Chignolin** (10 amino acids; 138 atoms), **Trp-cage** (284 atoms; 20 amino acids), and **BBA** (504 atoms; 28 amino acids). We define a hit as a trajectory where the final state reaches the metastable distribution $\pi_{ℬ}=\left\{R|\left\|\xi (R)-\xi \left(R_{ℬ}\right)\right\|<\right.0.75\}$, where ξ(R) are the top two time-lagged independent component analysis (TICA; Pérez-Hernández et al. (2013)) components. As shown in Fig. 4, EntangledSBM generates *diverse* transition paths across high-energy barriers that successfully reach the target state. We achieve a superior target hit percentage than all baselines across all proteins and a lower or comparable RMSD (Table 2). While we observe a higher average energy of transition state (ETS) compared to baselines, this can be attributed to the larger proportion and greater diversity of successful target-hitting paths. Given that the base dynamics $P^{0}$ rarely move beyond the initial metastable state, we show that EntangledSBM effectively learns the target bridge dynamics $P^{⋆}$ despite high energy barriers.

## 6 Conclusion

In this work, we present **Entangled Schrödinger Bridge Matching (EntangledSBM)**, a principled framework for learning the second-order dynamics of interacting multi-particle systems through entangled bias forces and an unconstrained cross-entropy objective. EntangledSBM captures dependencies between particle positions and velocities, enabling the modeling of complex dynamics across biological scales. For perturbation modeling, EntangledSBM reconstructs perturbed cell states while generalizing to divergent target states not seen during training, and for molecular dynamics (MD), it generates physically plausible transition paths for fast-folding proteins at an all-atom resolution.

### Limitations and Future Directions

Our experiments primarily demonstrate that integrating entangled velocities and a cross-entropy objective for path distribution matching enhances performance in modeling multi-particle systems. While we demonstrate that our unique parameterization enables performance gains across diverse systems across biological scales, it remains limited to a selected set of perturbations and small, fast-folding proteins. We envision our method generalizing across a broader range of drug-induced and genetic perturbations across diverse cell types and representations, larger proteins and biomolecules, as well as other multi-particle physical systems.

## Supplementary Material

1

## Figures and Tables

**Figure 1:**
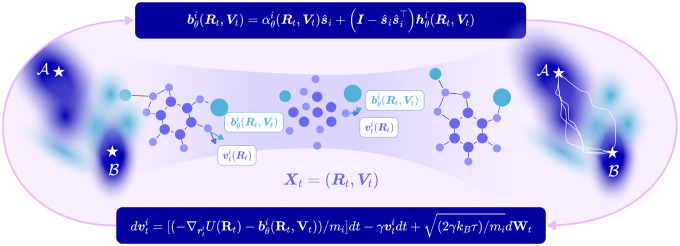
Entangled Schrödinger Bridge Matching. We consider the problem of simulating interacting multi-particle systems, where each particle’s velocity depends dynamically on the velocities of the other particles in the system, and introduce **EntangledSBM**, a framework that parameterizes an entangled bias force capturing the dynamic interactions between particles.

**Table 1:** Results for simulating cell cluster dynamics under Clonidine and Trametinib perturbation with EntangledSBM.

Clonidine Perturbation						
	Trained Target Distribution			Unseen Target Distribution		
Model	RBF-MMD (↓)	$W_{1}(↓)$	$W_{2}(↓)$	RBF-MMD (↓)	$W_{1}(↓)$	$W_{2}(↓)$
**Base Dynamics** (50 PCs)	0.677_±0.001_	5.947_±0.005_	6.015_±0.005_	0.784_±0.001_	8.217_±0.005_	8.384_±0.005_
**EntangledSBM w/o Velocity Conditioning**						
50 PCs	0.440_±0.000_	1.741_±0.003_	1.857_±0.004_	0.478_±0.000_	2.907_±0.006_	3.022_±0.006_
100 PCs	0.494_±0.000_	2.315_±0.004_	2.423_±0.004_	0.539_±0.000_	4.110_±0.004_	4.249_±0.003_
150 PCs	0.510_±0.000_	2.497_±0.006_	2.620_±0.006_	0.560_±0.000_	4.573_±0.006_	4.716_±0.007_
**EntangledSBM w/ CE**						
50 PCs	**0.401**_±0.000_	**0.342**_±0.002_	**0.400**_±0.001_	**0.419**_±0.000_	**0.538**_±0.013_	**0.705**_±0.030_
100 PCs	**0.455**_±0.000_	**0.953**_±0.025_	**1.015**_±0.025_	**0.500**_±0.001_	**0.899**_±0.006_	**1.055**_±0.008_
150 PCs	**0.478**_±0.000_	**0.753**_±0.008_	**0.826**_±0.007_	**0.506**_±0.000_	**0.700**_±0.009_	**0.811**_±0.011_

Trametinib Perturbation									
	Trained Target Distribution			Unseen Target Distribution 1			Unseen Target Distribution 2		
Method	RBF-MMD (↓)	$W_{1}(↓)$	$W_{2}(↓)$	RBF-MMD (↓)	$W_{1}(↓)$	$W_{2}(↓)$	RBF-MMD (↓)	$W_{1}(↓)$	$W_{2}(↓)$
**Base Dynamics**	0.938_±0.001_	7.637_±0.005_	7.653_±0.006_	0.900_±0.000_	7.766_±0.009_	7.877_±0.009_	0.754_±0.001_	1.201_±0.009_	1.455_±0.012_
**EntangledSBM w/o Velocity Conditioning**	0.449_±0.000_	1.506_±0.005_	1.544_±0.005_	0.476_±0.000_	2.116_±0.005_	2.197_±0.005_	0.480_±0.000_	0.505_±0.004_	0.627_±0.005_
**EntangledSBM w/ CE**	**0.428**_±0.000_	**0.392**_±0.005_	**0.434**_±0.006_	**0.409**_±0.000_	**0.453**_±0.008_	**0.561**_±0.009_	**0.451**_±0.000_	**0.394**_±0.003_	**0.469**_±0.004_

Best values for each PC dimension are **bolded**. We report RBF-MMD for all PCs and ${𝒲}_{1}$ and ${𝒲}_{2}$ distances of the top 2 PCs between ground truth and reconstructed clusters after 100 simulation steps and cluster size set to n=16. Mean and standard deviation of metrics from 5 independent simulations are reported. We simulate PCs d={50,100,150} for Clonidine and d=50 for Trametinib to cells sampled from the training target distribution and unseen target distributions, and compare against the learned bias force with no velocity conditioning.

**Table 2:** Transition path sampling benchmarks with EntangledSBM.

Protein	Alanine Dipeptide				Chignolin		
Method	RMSD (↓)	THP (↑)	ETS (↓)	Method	RMSD (↓)	THP (↑)	ETS (↓)
UMD †	1.19_±0.32_	6.25	–	UMD †	7.23_±0.93_	1.56	388.17
SMD (10K) †	0.86_±0.21_	7.81	812.47_±148.80_	SMD (10K) †	1.26_±0.31_	6.25	−527.95_±93.58_
SMD (20K) †	0.56_±0.27_	54.69	78.40_±12.76_	SMD (20K) †	**0.85**_±0.24_	34.38	179.52_±138.87_
PIPS (Force) †	0.66_±0.15_	43.75	28.17_±10.86_	PIPS (Force) †	4.66_±0.17_	0.00	–
TPS-DPS (Scalar) †	0.25_±0.20_	76.00	**22.79**_±13.57_	TPS-DPS (Scalar) †	1.17_±0.66_	59.38	−**780.18**_±216.93_
**EntangledSBM (Ours)**	**0.18**_±0.07_	**92.19**	47.91_±22.76_	**EntangledSBM (Ours)**	0.92_±0.13_	**64.06**	2825.61_±318.94_

Protein	Trp-cage				BBA		
Method	RMSD (↓)	THP (↑)	ETS (↓)	Method	RMSD (↓)	THP (↑)	ETS (↓)
UMD †	8.27_±1.13_	0.00	–	UMD †	10.81_±1.05_	0.00	-
SMD (10K) †	1.68_±0.23_	3.12	−312.54_±20.67_	SMD (10K) †	2.89_±0.32_	0.00	-
SMD (20K) †	1.20_±0.20_	42.19	−226.40_±85.59_	SMD (20K) †	1.66_±0.30_	26.56	−3104.95_±97.57_
PIPS (Force) †	7.47_±0.19_	0.00	-	PIPS (Force) †	9.84_±0.18_	0.00	-
TPS-DPS (Scalar) †	**0.76**_±0.12_	81.25	−**317.61**_±140.89_	TPS-DPS (Scalar) †	1.21_±0.09_	84.38	−**3801.68**_±139.38_
**EntangledSBM** (Ours)	1.04_±0.22_	**82.81**	765.74_±155.28_	**EntangledSBM** (Ours)	**0.84**_±0.08_	**96.88**	1453.80_±367.84_

Best values are **bolded**. All metrics are averaged over 64 paths. Unless specified in brackets, paths are generated at 300K for Alanine Dipeptide and Chignolin and 400K for the others. Hyperparameters and evaluation metrics are detailed in App E.

† denotes values taken from Seong et al. (2025).

## Data Availability

The codebase is freely accessible to the academic community at https://github.com/sophtang/EntangledSBM and https://huggingface.co/ChatterjeeLab/EntangledSBM.
