# Supplementary material for: Entangled Schrödinger Bridge Matching
Source: ArXiv. 2025 Nov 10:arXiv:2511.07406v1. Preprint. [Version 1] (PMC12642768)
Supplement: 1 [file NIHPP2511.07406V1-supplement-1.pdf]

Outline of Appendix

In App A, we provide an overview and discussion of related works in off-policy learning, Schrödinger bridge matching, and transition path sampling. App B provides the theoretical basis of stochastic optimal control (SOC) theory used in our work and a theoretical motivation for our entangled bias force. App C provides the theoretical formulations and proofs for EntangledSBM. We provide details on the experimental setup and training hyperparameters for the cell perturbation experiment in App D and the transition path sampling (TPS) experiment in App E. We further compare the log-variance (LV) divergence and cross-entropy (CE) objectives in App F. Finally, we provide the pseudocode for the training and inference of EntangledSBM in App G.

Notation In this work, we consider an n-particle system $\boldsymbol{X}_t = (\boldsymbol{R}_t, \boldsymbol{V}_t)$ where $\boldsymbol{R}_t \in \mathbb{R}^{n\times d}$ and $\boldsymbol{V}_t \in \mathbb{R}^{n\times d}$ denote the positions and velocities of the full system that evolves over the time horizon $t \in [0, T]$. The notation $\boldsymbol{X}$ indicates a random variable and $\boldsymbol{x}$ denotes a deterministic realization. Each particle i is defined by its position $\boldsymbol{r}_t^i \in \mathbb{R}^d$ and velocity $\boldsymbol{v}_t^i \in \mathbb{R}^d$ which lie in d-dimensional Euclidean space. The base dynamics evolve according to a potential energy function denoted $U(\boldsymbol{R}_t) : \mathbb{R}^{n\times d} \to \mathbb{R}$ and the biased dynamics evolve with an additional parameterized bias force $\boldsymbol{b}_\theta(\boldsymbol{R}_t, \boldsymbol{V}_t) : \mathbb{R}^{n\times d} \times \mathbb{R}^{n\times d} \to \mathbb{R}^{n\times d}$ that captures the implicit dependencies within the system and controls the base dynamics towards a target distribution $\pi_{\mathcal{B}}(\boldsymbol{R}_T)$ from a state in the initial distribution $\boldsymbol{R}_{\mathcal{A}} \in \pi_{\mathcal{A}}(\boldsymbol{R}_0)$. The corresponding path measures over trajectories $\boldsymbol{X}_{0:T} := (\boldsymbol{X}_t)_{t\in[0,T]}$ are denoted $\mathbb{P}^0$ for the base process and $\mathbb{P}^{b_\theta}$ for the biased process. The optimal path measure that solves the EntangledSB problem is denoted $\mathbb{P}^\star \equiv \mathbb{P}^{b^\star}$ with bias force $\boldsymbol{b}^\star(\boldsymbol{R}_t, \boldsymbol{V}_t)$.

A Related Works

Off-Policy Learning Importance-weighted cross-entropy (CE) objectives have previously been used to sample the target distribution $g^\star \propto hf_u$ by aligning a proposal distribution f_v to the intractable target with $\text{KL}(g^\star \| f_v)$ (Kappen & Ruiz, 2016). The main difference is that our approach aims to match a **controlled path measure** $\mathbb{P}^{b_\theta}$ with an energy-minimizing path measure $\mathbb{P}^\star$ rather than a static target distribution. Furthermore, we adapt our path CE objective to apply for discrete-time paths, similar to the approach taken by Seong et al. (2025), which discretizes the log-variance (LV) divergence (Nüsken & Richter, 2021). While the LV objective has theoretical optimality guarantees, it is non-convex in $\mathbb{P}^{b_\theta}$. Since the CE objective is convex in $\mathbb{P}^{b_\theta}$, it yields an ideal optimization landscape for θ and demonstrates improved performance as shown in Sec 5.1. We also highlight that our optimization scheme is a form of *off-policy* learning, as we minimize the objective with respect to the path distribution generated from an auxiliary control $\boldsymbol{v}$ which we set to the non-gradient-tracking bias force $\boldsymbol{v} := \bar{\boldsymbol{b}} = \texttt{stopgrad}(\boldsymbol{b}_\theta)$. This objective is inspired by recent work that leverages off-policy reinforcement learning in the discrete state space for discrete diffusion sampling (Zhu et al., 2025) and fine-tuning (Tang et al., 2025b).

Transition Path Sampling To overcome the challenge of generating feasible transition paths across high energy barriers for transition path sampling, several non-ML and ML-based approaches have been introduced (Bolhuis et al., 2002; Dellago et al., 1998; Vanden-Eijnden et al., 2010). Non-ML approaches often rely on collective variables (CVs), which reduce the dimensionality of the molecular conformation to a function of coordinates that are known to be involved in transition states (Hooft et al., 2021). These include steered MD (Schlitter et al., 1994; Izrailev et al., 1999), umbrella sampling (Torrie & Valleau, 1977; Kästner, 2011), meta-dynamics (Laio & Parrinello, 2002; Ensing et al., 2006; Branduardi et al., 2012; Bussi & Branduardi, 2015), adaptive biasing force (Comer et al., 2015), and on-the-fly probability-enhanced sampling (Invernizzi & Parrinello, 2020). To overcome the lack of well-understood CVs for large biomolecular systems, ML-based methods have been explored for determining or constructing CVs (Sultan & Pande, 2018; Rogal et al., 2019; Chen & Ferguson, 2018; Sun et al., 2022; Sipka et al., 2023b), but modeling transition paths at all-atom resolution is still desirable due to systems of increased complexity, and CVs are uncertain.

Several ML-based approaches for simulating transition paths have been developed (Singh & Limmer, 2023; Yan et al., 2022; Sipka et al., 2023a; Das et al., 2021). Notably, Path Integral Path Sampling (PIPS; Holdijk et al. (2023)) learns a bias force with stochastic optimal control theory, Doob's Lagrangian Du et al. (2024) defines an optimal transition path with a Lagrangian objective that solves

the Doob's h-transform, Transition Path Sampling with Diffusion Path Samplers (TPS-DPS; Seong et al. (2025)) trains a bias force with an off-policy log-variance objective, and Action Minimization with Onsager-Machlup (OM) Functional (Raja et al., 2025) generates a discrete interpolation between endpoints by minimizing the OM functional. Furthermore, Blessing et al. (2025) introduces a trust-region-constrained SOC optimization algorithm, which is applied to TPS. While these approaches demonstrate the promise of ML-based TPS, their applicability remains limited to small-scale biomolecular systems (Alanine Dipeptide, tetrapeptides, etc.) for all-atom simulation (Holdijk et al., 2023; Du et al., 2024) or require coarse-grained representations when scaling up to larger fast-folding proteins (Raja et al., 2025). We directly compare our method against Seong et al. (2025) and Blessing et al. (2025), and achieve state-of-the-art performance on all-atom simulations of larger, fast-folding proteins, and Blessing et al. (2025). Furthermore, we highlight that our framework extends beyond molecular dynamics (MD) simulations, with applications to single-cell simulation and generalization to unseen target distributions.

Modeling Cell Dynamics Under Perturbation Predicting the dynamics of heterogeneous cell populations under perturbations such as treatment with a drug candidate, gene editing, and knockouts, or protein expression, has critical applications in drug discovery and predictive medicine (Shalem et al., 2014; Kramme et al., 2021; Dixit et al., 2016; Gavriilidis et al., 2024; Zhang et al., 2025a; Kobayashi et al., 2022; Smela et al., 2023a; Pierson Smela et al., 2025; Yeo et al., 2021). Generative modeling frameworks including flow matching (Zhang et al., 2025b; Rohbeck et al., 2025; Atanackovic et al., 2024; Tong et al., 2024; Wang et al., 2025), Schrödinger Bridge Matching (Alatkar & Wang, 2025; Tong et al., 2024; Kapuśniak et al., 2024; Tang et al., 2025a), and stochastic optimal control (Zhang et al., 2025c;d) have enabled significant advances in parameterizing velocities that evolve dynamically over time on non-linear energy manifolds by learning on static snapshots across the trajectory. However, these models often require *explicit training on a specific target distribution* and fail to generalize to unseen target distributions at inference, especially if they diverge from those seen in the training data, which limits their scalability to cell types and perturbations that are not seen during training. Furthermore, many of these approaches simulate cells as populations of particles that evolve independently from each other, which fails to capture the diverse intercellular signaling and interactions that occur naturally and under perturbations. Although there have been some early developments in learning these interactions from data (Zhang et al., 2025d), they operate under the mean-field assumption, where each cell takes the average effect of all surrounding cells rather than simulating individual pairwise interactions. This assumption does not account for the complex interactions between cells and limits its applicability to heterogeneous cell populations.

B Extended Theoretical Preliminaries

In this section, we provide relevant theoretical background in stochastic optimal control (SOC) theory for path measures. We use the terms *bias force* and *optimal control* interchangeably, denoted with $\boldsymbol{b}$, which is less common in general SOC literature where $\boldsymbol{u}$ is often used. For a more comprehensive analysis of SOC theory, we refer the reader to Nüsken & Richter (2021).

B.1 Stochastic Optimal Control

Controlled Path Measures First, we consider the controlled path measure $\mathbb{P}^b$ defined by the SDE:

$$d\boldsymbol{X}_t = [\boldsymbol{f}(\boldsymbol{X}_t, t) + \boldsymbol{\Sigma}\boldsymbol{b}(\boldsymbol{X}_t, t)]dt + \boldsymbol{\Sigma}d\boldsymbol{W}_t \tag{18}$$

where $\boldsymbol{b} : [0, T] \times \mathcal{X} \to \mathcal{X} \in \mathcal{U}$ is known as the **control**[1] that tilts the path measure from the unconditional dynamics $\mathbb{P}^0$. Now, we will define **Radon-Nikodym derivative (RND)** of the path measures corresponding to the controlled and unconditional SDEs, which is crucial for defining our objective.

[1]$\mathcal{U}$ is the set of admissible controls continuously differentiable in $(\boldsymbol{x}, t)$ with bounded linear growth in $\boldsymbol{x}$

Lemma B.1 (Radon-Nikodym Derivative). *Given a pair of SDEs with and without a control drift $\boldsymbol{b}(\boldsymbol{X}_t, t)$ on $t \in [0, T]$ with the same diffusion $\boldsymbol{\Sigma}$ defined as*

$$\mathbb{P}^0 : d\boldsymbol{X}_t = \boldsymbol{f}(\boldsymbol{X}_t, t)dt + \boldsymbol{\Sigma} d\boldsymbol{W}_t, \qquad \boldsymbol{X}_0 = \boldsymbol{x}_0 \tag{19}$$

$$\mathbb{P}^b : d\boldsymbol{X}_t^b = [\boldsymbol{f}(\boldsymbol{X}_t, t) + \boldsymbol{\Sigma}\boldsymbol{b}(\boldsymbol{X}_t, t)]\, dt + \boldsymbol{\Sigma} d\boldsymbol{W}_t, \qquad \boldsymbol{X}_0^b = \boldsymbol{x}_0 \tag{20}$$

Then, the Radon-Nikodym derivative satisfies

$$\log \frac{\mathrm{d}\mathbb{P}^b}{\mathrm{d}\mathbb{P}^0}(\boldsymbol{X}_{0:T}) = \int_0^T (\boldsymbol{b}^\top \boldsymbol{\Sigma}^{-1})(\boldsymbol{X}_t, t) d\boldsymbol{X}_t - \int_0^T (\boldsymbol{\Sigma}^{-1}\boldsymbol{f} \cdot \boldsymbol{b})(\boldsymbol{X}_t, t)dt - \frac{1}{2}\int_0^T \|\boldsymbol{b}(\boldsymbol{X}_t, t)\|^2 dt$$

or equivalently:

$$\log \frac{\mathrm{d}\mathbb{P}^b}{\mathrm{d}\mathbb{P}^0}(\boldsymbol{X}_{0:T}) = \int_0^T \boldsymbol{b}(\boldsymbol{X}_t, t)^\top d\boldsymbol{W}_t - \frac{1}{2}\int_0^T \|\boldsymbol{b}(\boldsymbol{X}_t, t)\|^2 dt \tag{21}$$

Proof. First, we use Girsanov's theorem to get the Radon-Nikodym derivative with respect to the zero-drift reference path measure $\mathbb{Q}$ defined by the SDE $d\boldsymbol{X}_t = \boldsymbol{\Sigma}(\boldsymbol{X}_t, t)d\boldsymbol{W}_t$, we have

$$\log \frac{\mathbb{P}^0}{\mathbb{Q}}(\boldsymbol{X}_{0:T}) = \int_0^T \big(\boldsymbol{f}(\boldsymbol{X}_t, t) \cdot \boldsymbol{\Sigma}^{-2}(\boldsymbol{X}_t, t)\big)\, d\boldsymbol{X}_t - \frac{1}{2}\int_0^T \big(\boldsymbol{f} \cdot \boldsymbol{\Sigma}^{-2}\boldsymbol{f}\big)\,(\boldsymbol{X}_t, t)dt \tag{22}$$

and for the controlled path measure $\mathbb{P}^b$, we have

$$\begin{aligned}\log \frac{\mathbb{P}^b}{\mathbb{Q}}(\boldsymbol{X}_{0:T}) &= \int_0^T (\boldsymbol{f} + \boldsymbol{\Sigma}\boldsymbol{b})(\boldsymbol{X}_t, t) \cdot \boldsymbol{\Sigma}^{-2}(\boldsymbol{X}_t, t) d\boldsymbol{X}_t \\ &\quad - \frac{1}{2}\int_0^T \big((\boldsymbol{f} + \boldsymbol{\Sigma}\boldsymbol{b}) \cdot \boldsymbol{\Sigma}^{-2}(\boldsymbol{f} + \boldsymbol{\Sigma}\boldsymbol{b})\big)\,(\boldsymbol{X}_t, t)dt\end{aligned} \tag{23}$$

Then, using the identity

$$\log \frac{\mathrm{d}\mathbb{P}^b}{\mathrm{d}\mathbb{P}^0} = \log \frac{\mathrm{d}\mathbb{P}^b}{\mathrm{d}\mathbb{Q}}\frac{\mathrm{d}\mathbb{Q}}{\mathrm{d}\mathbb{P}^0} = \log \frac{\mathrm{d}\mathbb{P}^b}{\mathrm{d}\mathbb{Q}} + \log \frac{\mathrm{d}\mathbb{Q}}{\mathrm{d}\mathbb{P}^0} = \log \frac{\mathrm{d}\mathbb{P}^b}{\mathrm{d}\mathbb{Q}} - \log \frac{\mathrm{d}\mathbb{P}^0}{\mathrm{d}\mathbb{Q}} \tag{24}$$

Substituting in (22) and (23) and canceling terms, we get

$$\log \frac{\mathrm{d}\mathbb{P}^b}{\mathrm{d}\mathbb{P}^0}(\boldsymbol{X}_{0:T}) = \int_0^T (\boldsymbol{b}^\top \boldsymbol{\Sigma}^{-1})(\boldsymbol{X}_t, t) d\boldsymbol{X}_t - \int_0^T (\boldsymbol{\Sigma}^{-1}\boldsymbol{f} \cdot \boldsymbol{b})(\boldsymbol{X}_t, t)dt - \frac{1}{2}\int_0^T \|\boldsymbol{b}(\boldsymbol{X}_t, t)\|^2 dt \tag{25}$$

Equivalently, since we can write $d\boldsymbol{W}_t = \boldsymbol{\Sigma}^{-1}(\boldsymbol{X}_t, t)(d\boldsymbol{X}_t + \boldsymbol{b}(\boldsymbol{X}_t, t)dt)$, we can write

$$\log \frac{\mathrm{d}\mathbb{P}^b}{\mathrm{d}\mathbb{P}^0}(\boldsymbol{X}_{0:T}) = \int_0^T \boldsymbol{b}(\boldsymbol{X}_t, t)^\top d\boldsymbol{W}_t - \frac{1}{2}\int_0^T \|\boldsymbol{b}(\boldsymbol{X}_t, t)\|^2 dt \tag{26}$$

which concludes the proof. □

Stochastic Optimal Control The reward-guided **stochastic optimal control (SOC)** problem aims to determine the **optimal control** $\boldsymbol{b}^\star = \min_{\boldsymbol{b} \in \mathcal{U}} J(\boldsymbol{b}; \boldsymbol{x}, t)$ that minimizes the **cost functional** $J(\boldsymbol{b}_\theta; \boldsymbol{x}, t)$ with reward $r : \mathcal{X} \to \mathbb{R}$ given by

$$J(\boldsymbol{b}; \boldsymbol{x}, t) = \mathbb{E}_{p_t}\left[\int_t^T \left(\boldsymbol{f}(\boldsymbol{X}_s, s) + \frac{1}{2}\|\boldsymbol{b}(\boldsymbol{X}_s, s)\|^2\right) ds - r(\boldsymbol{X}_T) \middle| \boldsymbol{X}_t = \boldsymbol{x}\right] \tag{27}$$

which measures the *cost-to-go* from the current state $\boldsymbol{X}_t = \boldsymbol{x}$ at time t under the controlled dynamics defined in (18) to the terminal state $\boldsymbol{X}_T$. The infimum of the cost functional is defined as

$$J(\boldsymbol{b}^\star; \boldsymbol{x}, t) = \inf_{\boldsymbol{b} \in \mathcal{U}} J(\boldsymbol{b}; \boldsymbol{x}, t) =: V_t(\boldsymbol{x}) \tag{28}$$

which is also referred to as the *value function* $V_t(\boldsymbol{x})$. To derive the path integral representation of the optimal control $\boldsymbol{b}^\star$, we introduce the *work functional* which computes the running cost subtracted by the terminal reward of a state path $\boldsymbol{X}_{0:T}$,

$$\mathcal{W}(\boldsymbol{X}_{t:T}, t) := \int_t^T \boldsymbol{f}(\boldsymbol{X}_s, s)ds - r(\boldsymbol{X}_T) \tag{29}$$

We can now define a series of statements that connect the optimal drift $\boldsymbol{b}^\star$ and the value function V_t.

Theorem B.1. *Let V_t be the value function and $\boldsymbol{b}^\star$ be the optimal control. Then, the following are true:*

(a) The optimal control satisfies $\boldsymbol{b}^\star(\boldsymbol{x}) = -\boldsymbol{\Sigma}^\top \nabla_{\boldsymbol{x}} V_t(\boldsymbol{x})$

(b) The Radon-Nikodym derivative of the optimal path measure $\mathbb{P}^\star$ can be defined by taking state paths from the unconditional path measure $\mathbb{P}^0$ and reweighting them with the output of the work functional $\mathcal{W}(\boldsymbol{X}_{0:T}, 0)$.

$$\frac{\mathrm{d}\mathbb{P}^\star}{\mathrm{d}\mathbb{P}^0}(\boldsymbol{X}_{0:T}) = \frac{1}{Z} e^{-\mathcal{W}(\boldsymbol{X}_{0:T},0)}, \quad Z = \mathbb{E}_{\boldsymbol{X}_{0:T}\sim\mathbb{P}^0}\left[e^{-\mathcal{W}(\boldsymbol{X}_{0:T},0)}\right] \tag{30}$$

(c) For any $(\boldsymbol{x}, t) \in \mathcal{X} \times [0, T]$, the value function can be written in path integral form by taking the expectation over the uncontrolled path measure $\mathbb{P}^0$ as

$$V_t(\boldsymbol{x}, t) = -\log \mathbb{E}\left[e^{-\mathcal{W}(\boldsymbol{X}_{t:T},t)} \middle| \boldsymbol{X}_t = \boldsymbol{x}\right] \tag{31}$$

(d) Combining (a) and (c), we can write the optimal control as

$$\boldsymbol{b}^\star(\boldsymbol{x}) = \boldsymbol{\Sigma}^\top \nabla_{\boldsymbol{x}} \log \mathbb{E}_{\boldsymbol{X}_{t:T}\sim\mathbb{P}^0}\left[e^{-\mathcal{W}(\boldsymbol{X}_{t:T},t)} \middle| \boldsymbol{X}_t = \boldsymbol{x}\right] \tag{32}$$

While Theorem B.1 provides a closed-form solution to the optimal control $\boldsymbol{b}^\star$, it remains impractical to compute as $\mathbb{P}^0$ contains an infinite number of paths from any point $\boldsymbol{x}$. This problem motivates parameterizing $\boldsymbol{b}^\star$ with a neural network $\boldsymbol{b}_\theta$. The natural objective for obtaining an accurate approximation of $\boldsymbol{b}^\star$ that induces the path measure $\mathbb{P}^\star$ is the KL-divergence $\mathcal{L}(\mathbb{P}^\star, \mathbb{P}^{b_\theta}) := D_{\mathrm{KL}}(\mathbb{P}^{b_\theta} \| \mathbb{P}^\star)$ as its minimizer is exactly $\mathbb{P}^\star$.

However, taking the gradient $\nabla_\theta D_{\mathrm{KL}}(\mathbb{P}^{b_\theta} \| \mathbb{P}^\star)$ requires differentiating through the full stochastic trajectories $\boldsymbol{X}_{0:T} \sim \mathbb{P}^{b_\theta}$ generated by the Euler-Maruyama SDE solver due to the expectation over $\mathbb{P}^{b_\theta}$, resulting in a significant computational bottleneck. To overcome this bottleneck, previous work (Nüsken & Richter, 2021; Seong et al., 2025) leverages alternative path-measure objectives that do not involve expectations over $\mathbb{P}^{b_\theta}$ while preserving optimality guarantees. We focus on two objectives: the **log-variance divergence** used in Seong et al. (2025) and the **cross-entropy objective**, which we formalize in the present work for solving the EntangledSB problem.

Log-Variance Objective Here, we describe the **log-variance (LV) divergence** which is introduced in Nüsken & Richter (2021) and applied to transition path sampling (TPS) in TPS-DPS (Seong et al., 2025). Given the path measure $\mathbb{P}^{b_\theta}$ generated with the parameterized bias force $\boldsymbol{b}_\theta$ and the target path measure $\mathbb{P}^\star$, the log-variance divergence $\mathcal{L}_{\mathrm{LV}}$ is defined as

$$\mathcal{L}_{\mathrm{LV}}(\mathbb{P}^\star, \mathbb{P}^{b_\theta}) := \mathrm{Var}_{\mathbb{P}^v}\left[\log \frac{\mathrm{d}\mathbb{P}^\star}{\mathrm{d}\mathbb{P}^{b_\theta}}\right] = \mathbb{E}_{\mathbb{P}^v}\left[\left(\log \frac{\mathrm{d}\mathbb{P}^\star}{\mathrm{d}\mathbb{P}^{b_\theta}} - \mathbb{E}_{\mathbb{P}^v}\left[\log \frac{\mathrm{d}\mathbb{P}^\star}{\mathrm{d}\mathbb{P}^{b_\theta}}\right]\right)^2\right] \tag{33}$$

where $\mathbb{P}^v$ can be defined as any arbitrary path measure generated from a control $\boldsymbol{v}$. To allow simulated trajectories to be used across multiple training iterations, it is common to define $\boldsymbol{v}$ as the bias force with frozen parameters $\boldsymbol{v} = \bar{\boldsymbol{b}} := \texttt{stopgrad}(\boldsymbol{b}_\theta)$. Using Lemma B.1, we define $\mathcal{L}_{\mathrm{LV}}(\mathbb{P}^\star, \mathbb{P}^{b_\theta}) = \mathbb{E}_{\mathbb{P}^v}[(\mathcal{F}_{\boldsymbol{b}_\theta,\boldsymbol{v}} - \mathbb{E}_{\mathbb{P}^v}[\mathcal{F}_{\boldsymbol{b}_\theta,\boldsymbol{v}}])^2]$, where $\mathcal{F}_{\boldsymbol{b}_\theta,\boldsymbol{v}}$ is defined as

$$\begin{aligned}\mathcal{F}_{\boldsymbol{b}_\theta,\boldsymbol{v}} &:= \log \frac{\mathrm{d}\mathbb{P}^\star}{\mathrm{d}\mathbb{P}^{b_\theta}} = \log \frac{\mathrm{d}\mathbb{P}^\star}{\mathrm{d}\mathbb{P}^0}\frac{\mathrm{d}\mathbb{P}^0}{\mathrm{d}\mathbb{P}^{b_\theta}} \\ &= r(\boldsymbol{X}_T) + \frac{1}{2}\int_0^T \|\boldsymbol{b}_\theta(\boldsymbol{X}_t)\|^2 dt - \int_0^T (\boldsymbol{b}_\theta^\top \boldsymbol{v})(\boldsymbol{X}_t) dt - \int_0^T \boldsymbol{b}_\theta^\top d\boldsymbol{W}_t\end{aligned} \tag{34}$$

using similar justification as in App C.4. Since the expectation $\mathbb{E}_{\mathbb{P}^v}[\mathcal{F}_{\boldsymbol{b}_\theta,\boldsymbol{v}}]$ is computationally intractable, Seong et al. (2025) introduces a scalar parameter w that is jointly optimized with θ such that $\arg\min_w \mathcal{L}_{\mathrm{LV}}(\theta, w) = \mathbb{E}_{\mathbb{P}^v}[\mathcal{F}_{\boldsymbol{b}_\theta,\boldsymbol{v}}]$. Substituting in w for $\mathbb{E}_{\mathbb{P}^v}[\mathcal{F}_{\boldsymbol{b}_\theta,\boldsymbol{v}}]$ and taking the discretization over K steps, the LV objective becomes

$$\mathcal{L}_{\mathrm{LV}}(\theta, w) = \mathbb{E}_{\boldsymbol{X}_{0:K}\sim\mathbb{P}^v}\left[(\mathcal{F}_{\boldsymbol{b}_\theta,\boldsymbol{v}}(\boldsymbol{X}_{0:K}) - w)^2\right] = \mathbb{E}_{\boldsymbol{x}_{0:K}\sim\mathbb{P}^v}\left[\left(\log \frac{p^0(\boldsymbol{x}_{0:K})\exp(r(\boldsymbol{X}_K))}{p^{b_\theta}(\boldsymbol{x}_{0:K})} - w\right)^2\right]$$

where $\boldsymbol{v} = \bar{\boldsymbol{b}} := \texttt{stopgrad}(\boldsymbol{b}_\theta)$.

B.2 JUSTIFICATION FOR ENTANGLED BIAS FORCES

Proposition B.1 (Monotone Optimality of Entangled Bias Forces)**.** *Let $\mathcal{F}_{ind}$ be the hypothesis class of bias forces that depend only on particle positions $\boldsymbol{b}_\theta^i := \boldsymbol{b}_\theta^i(\boldsymbol{R}_t)$ and $\mathcal{F}_{ent}$ be the hypothesis class of entangled bias forces $\boldsymbol{b}_\theta^i := \boldsymbol{b}_\theta^i(\boldsymbol{R}_t, \boldsymbol{V}_t)$. Then, under the cross-entropy objective,*

$$\inf_{\boldsymbol{b}_\theta \in \mathcal{F}_{ent}} \mathcal{L}_{CE}(\boldsymbol{b}_\theta) \leq \inf_{\boldsymbol{b}_\theta \in \mathcal{F}_{ind}} \mathcal{L}_{CE}(\boldsymbol{b}_\theta) \tag{35}$$

with strict improvement when the optimal control is non-factorizable.

Proof. Let $\mathbb{P}^\star$ denote the optimal path measure that solves the EntangledSB problem from (3.1) and $\mathbb{P}^{b_\theta}$ be the path measure induced by the bias force $\boldsymbol{b}_\theta$. Let $\mathcal{F}_{\text{ind}}$ be the hypothesis class of bias forces independent of the full system velocities $\boldsymbol{b}_\theta^i := \boldsymbol{b}_\theta^i(\boldsymbol{R}_t)$ and $\mathcal{F}_{\text{ent}}$ be the hypothesis class of entangled bias forces $\boldsymbol{b}_\theta^i := \boldsymbol{b}_\theta^i(\boldsymbol{R}_t, \boldsymbol{V}_t)$. Clearly, we have $\mathcal{F}_{\text{ind}} \subseteq \mathcal{F}_{\text{ent}}$. It follows that

$$\{\mathbb{P}^{b_\theta} : \boldsymbol{b}_\theta \in \mathcal{F}_{\text{ind}}\} \subseteq \{\mathbb{P}^{b_\theta} : \boldsymbol{b}_\theta \in \mathcal{F}_{\text{ent}}\} \tag{36}$$

Taking the infima over the KL-divergence functional $D_{\text{KL}}(\mathbb{P}^\star \| \cdot) \geq 0$ over a larger set of functions cannot increase the value.

$$\inf_{\boldsymbol{b}_\theta \in \mathcal{F}_{\text{ent}}} D_{\text{KL}}(\mathbb{P}^\star \| \mathbb{P}^{b_\theta}) \leq \inf_{\boldsymbol{b}_\theta \in \mathcal{F}_{\text{ind}}} D_{\text{KL}}(\mathbb{P}^\star \| \mathbb{P}^{b_\theta}) \tag{37}$$

Furthermore, if $\mathbb{P}^\star \notin \overline{\{\mathbb{P}^{b_\theta} : \boldsymbol{b}_\theta \in \mathcal{F}_{\text{ind}}\}}$ which denotes the closure of the set of path measures induced by $\mathcal{F}_{\text{ind}}$ and there exists $\boldsymbol{b}^\star \in \overline{\mathcal{F}_{\text{ent}}}$ where $\mathbb{P}^{\boldsymbol{b}^\star} = \mathbb{P}^\star$, then we have

$$\inf_{\boldsymbol{b}_\theta \in \mathcal{F}_{\text{ent}}} D_{\text{KL}}(\mathbb{P}^\star \| \mathbb{P}^{b_\theta}) = 0 \quad \text{while} \quad \inf_{\boldsymbol{b}_\theta \in \mathcal{F}_{\text{ind}}} D_{\text{KL}}(\mathbb{P}^\star \| \mathbb{P}^{b_\theta}) > 0 \tag{38}$$

yielding strict improvement. By Proposition 4.2, we have that $\mathbb{P}^{b^\star} = \mathbb{P}^\star$ is the unique minimizer of $D_{\text{KL}}(\mathbb{P}^\star \| \cdot)$. □

C THEORETICAL PROOFS

For notational simplicity, we will drop the explicit dependence on $(\boldsymbol{R}_t, \boldsymbol{V}_t)$ and simply denote the stochastic path of the system as $(\boldsymbol{X}_t)_{t \in [0,T]}$.

C.1 PROOF OF PROPOSITION 3.1

Lemma C.1. *The bias force $\boldsymbol{b}^\star$ that minimizes the SOC objective $J(\boldsymbol{b}_\theta; \boldsymbol{x}, t)$ generates the path measure $\mathbb{P}^b$ that minimizes the KL-divergence with the optimal tilted path measure $\mathbb{P}^\star$ that satisfies*

$$\mathbb{P}^\star = \frac{1}{Z} \mathbb{P}^0 e^{r(\boldsymbol{X}_T)}, \quad \textit{where} \;\; Z = \mathbb{E}_{\mathbb{P}_T^0}\left[e^{r(\boldsymbol{X}_T)}\right] \tag{39}$$

where $\mathbb{P}^0$ is the base path measure.

Proof. Let the dynamics of $\boldsymbol{X}_t = (\boldsymbol{R}_t, \boldsymbol{V}_t)$ under the base path measure $\mathbb{P}^0$ be defined as

$$d\boldsymbol{X}_t = \boldsymbol{f}(\boldsymbol{X}_t)dt + \boldsymbol{\Sigma} d\boldsymbol{W}_t \tag{40}$$

and the dynamics under the biased path measure $\mathbb{P}^{b_\theta}$ be defined as

$$d\boldsymbol{X}_t = \left[\boldsymbol{f}(\boldsymbol{X}_t) + \boldsymbol{\Sigma} \boldsymbol{b}_\theta(\boldsymbol{X}_t)\right] dt + \boldsymbol{\Sigma} d\boldsymbol{W}_t \tag{41}$$

By Lemma B.1, we have that the logarithm of the Radon-Nikodym derivative of the biased measure $\mathbb{P}^{b_\theta}$ with respect to the base measure $\mathbb{P}^0$ is given by

$$\log \frac{\mathrm{d}\mathbb{P}^{b_\theta}}{\mathrm{d}\mathbb{P}^0}(\boldsymbol{X}_{0:T}) = \int_0^T \boldsymbol{b}_\theta(\boldsymbol{X}_t)^\top d\boldsymbol{W}_t - \frac{1}{2} \int_0^T \|\boldsymbol{b}_\theta(\boldsymbol{X}_t)\|^2 dt \tag{42}$$

Now, taking the expectation with respect to $\mathbb{P}^{b_\theta}$, we derive the expression for the KL-divergence

$$\begin{aligned}
D_{\mathrm{KL}}(\mathbb{P}^{b_\theta}\|\mathbb{P}^0) &= \mathbb{E}_{\mathbb{P}^{b_\theta}}\left[\log\frac{\mathrm{d}\mathbb{P}^{b_\theta}}{\mathrm{d}\mathbb{P}^0}\right] \\
&= \mathbb{E}_{\mathbb{P}^{b_\theta}}\left[\int_0^T \boldsymbol{b}_\theta(\boldsymbol{X}_t)^\top d\boldsymbol{W}_t - \frac{1}{2}\int_0^T \|\boldsymbol{b}_\theta(\boldsymbol{X}_t)\|^2 dt\right] \\
&= \mathbb{E}_{\mathbb{P}^{b_\theta}}\left[\int_0^T \boldsymbol{b}_\theta(\boldsymbol{X}_t)^\top (d\boldsymbol{W}_t^b + \boldsymbol{b}_\theta(\boldsymbol{X}_t)dt) - \frac{1}{2}\int_0^T \|\boldsymbol{b}_\theta(\boldsymbol{X}_t)\|^2 dt\right] \\
&= \underbrace{\mathbb{E}_{\mathbb{P}^{b_\theta}}\left[\int_0^T \boldsymbol{b}_\theta(\boldsymbol{X}_t)^\top d\boldsymbol{W}_t^b\right]}_{=0} + \mathbb{E}_{\mathbb{P}^{b_\theta}}\left[\int_0^T \|\boldsymbol{b}_\theta(\boldsymbol{X}_t)\|^2 dt - \frac{1}{2}\int_0^T \|\boldsymbol{b}_\theta(\boldsymbol{X}_t)\|^2 dt\right] \\
&= \frac{1}{2}\mathbb{E}_{\mathbb{P}^{b_\theta}}\int_0^T \|\boldsymbol{b}_\theta(\boldsymbol{X}_t)\|^2 dt
\end{aligned} \tag{43}$$

Substituting this into the SOC objective in (3.1), we get

$$\begin{aligned}
J(\boldsymbol{b}_\theta) &= \mathbb{E}_{\mathbb{P}^{b_\theta}}\left[\int_0^T \frac{1}{2}\|\boldsymbol{b}_\theta(\boldsymbol{X}_t)\|^2 dt - r(\boldsymbol{X}_T)\right] \\
&= D_{\mathrm{KL}}(\mathbb{P}^{b_\theta}\|\mathbb{P}^0) - \mathbb{E}_{\mathbb{P}^{b_\theta}}[r(\boldsymbol{X}_T)]
\end{aligned} \tag{44}$$

By adding $\log Z$ on both sides, we do not change the minimizer of $J(\boldsymbol{b}_\theta)$ and the SOC objective becomes the KL-divergence between the controlled path measure $\mathbb{P}^{b_\theta}$ and the optimal path measure $\mathbb{P}^\star$:

$$\begin{aligned}
J(\boldsymbol{b}_\theta) + \log Z &= D_{\mathrm{KL}}(\mathbb{P}^{b_\theta}\|\mathbb{P}^0) - \mathbb{E}_{\mathbb{P}^{b_\theta}}[r(\boldsymbol{X}_T)] + \log Z \\
&= \underbrace{\mathbb{E}_{\mathbb{P}^{b_\theta}}\left[\log\frac{\mathrm{d}\mathbb{P}^{b_\theta}}{\mathrm{d}\mathbb{P}^0} - r(\boldsymbol{X}_T) + \log Z\right]}_{D_{\mathrm{KL}}(\mathbb{P}^{b_\theta}\|\mathbb{P}^\star)}
\end{aligned} \tag{45}$$

This shows that the minimizer $\boldsymbol{b}^\star$ to the SOC objective also generates the path measure $\mathbb{P}^{b^\star}$ that is closest in KL-divergence to the optimal path measure $\mathbb{P}^\star$. Therefore, the optimal control solution satisfies

$$\log\frac{\mathrm{d}\mathbb{P}^\star}{\mathrm{d}\mathbb{P}^0}(\boldsymbol{X}_{0:T}) = r(\boldsymbol{X}_T) - \log Z \iff \mathbb{P}^\star = \frac{1}{Z}\mathbb{P}^0 e^{r(\boldsymbol{X}_T)} \tag{46}$$

which concludes our proof. □

Proposition 3.1 (Solving EntangledSB with Stochastic Optimal Control)**.** *We can solve the EntangledSB problem with the stochastic optimal control (SOC) objective given by*

$$\boldsymbol{b}^\star = \arg\min_{\boldsymbol{b}_\theta}\mathbb{E}_{\boldsymbol{X}_{0:T}\sim\mathbb{P}^{b_\theta}}\left[\int_0^T \frac{1}{2}\|\boldsymbol{b}_\theta(\boldsymbol{R}_t, \boldsymbol{V}_t)\|^2 dt - r(\boldsymbol{X}_T)\right] \quad \textit{s.t.} \quad (6) \tag{8}$$

where $r(\boldsymbol{X}_T) := \log\pi_{\mathcal{B}}(\boldsymbol{R}_T)$ is the terminal reward that measures the log-probability under the target distribution.

We aim to recover the optimal path measure $\mathbb{P}^\star$ that minimizes the KL divergence from the base dynamics $\mathbb{P}^0$ while matching the terminal distribution $\pi_{\mathcal{B}}$ defined as

$$\mathbb{P}^\star(\boldsymbol{X}_{0:T}) = \frac{1}{Z}\mathbb{P}^0\pi_{\mathcal{B}}(\boldsymbol{X}_T) \tag{47}$$

From Lemma C.1, we have that the bias force $\boldsymbol{b}_\theta$ that minimizes the SOC objective defined as

$$\boldsymbol{b}^\star = \arg\min_{\boldsymbol{b}_\theta}\mathbb{E}_{\boldsymbol{X}_{0:T}\sim\mathbb{P}^{b_\theta}}\left[\int_0^T \frac{1}{2}\|\boldsymbol{b}_\theta(\boldsymbol{X}_t)\|^2 dt - r(\boldsymbol{X}_T)\right] \tag{48}$$

also generates the path measure $\mathbb{P}^{b^\star}$ that minimizes the KL-divergence $D_{\text{KL}}(\mathbb{P}^{b_\theta}\|\mathbb{P}^\star)$ with the optimal path measure $\mathbb{P}^\star$ defined as

$$\mathbb{P}^\star = \frac{1}{Z}\mathbb{P}^0 e^{r(\boldsymbol{X}_T)} \tag{49}$$

Therefore, setting $r(\boldsymbol{X}_T) := \log \pi_{\mathcal{B}}(\boldsymbol{X}_T)$ recovers the solution to the EntangledSB problem. □

C.2 Proof of Proposition 4.1

Proposition 4.1 (Non-Increasing Distance from Target Distribution)**.** *For small enough Δt, the distance from some target state $\boldsymbol{R}_{\mathcal{B}} \in \pi_{\mathcal{B}}$ state after an update with the bias force $\boldsymbol{b}^i_\theta(\boldsymbol{R}_t, \boldsymbol{V}_t)$ defined in (10) is non-increasing, such that*

$$\exists \boldsymbol{R}_{\mathcal{B}} \in \pi_{\mathcal{B}} \ \text{ s.t. } \ \|\boldsymbol{R}_{t+\Delta t} - \boldsymbol{R}_{\mathcal{B}}\| \leq \|\boldsymbol{R}_t - \boldsymbol{R}_{\mathcal{B}}\| \tag{11}$$

where $\boldsymbol{R}_{t+\Delta t} = \boldsymbol{R}_t + (\boldsymbol{b}^i_\theta(\boldsymbol{R}_t, \boldsymbol{V}_t)/m_i)\Delta t$.

Proof. We aim to show that our parameterization of the bias force in (10) ensures that the distance between the current position $\boldsymbol{R}_t$ and some target state $\boldsymbol{R}_T \sim \pi_{\mathcal{B}}$ in the target distribution is *non-increasing*. For each atom $i \in \{1, \ldots, n\}$, we define the bias force as

$$\boldsymbol{b}^i_\theta := \alpha^i_\theta \hat{\boldsymbol{s}}_i + \left(\boldsymbol{I} - \hat{\boldsymbol{s}}_i\hat{\boldsymbol{s}}_i^\top\right)\boldsymbol{h}^i_\theta, \qquad \alpha^i_\theta \geq 0, \ \ \hat{\boldsymbol{s}}_i = \frac{\nabla_{\boldsymbol{r}^i_t}\log \pi_{\mathcal{B}}}{\|\nabla_{\boldsymbol{r}^i_t}\log \pi_{\mathcal{B}}\|} \tag{50}$$

After the update in the direction of the potential energy $-\nabla_{\boldsymbol{r}^i_t}U(\boldsymbol{R}_t)$, the Euler update to the position with time step Δt using the bias force is

$$\boldsymbol{r}^i_{t+\Delta t} = \boldsymbol{r}^i_t + \frac{\Delta t}{m_i}\left(\alpha^i_\theta\hat{\boldsymbol{s}}_i + \left(\boldsymbol{I} - \hat{\boldsymbol{s}}_i\hat{\boldsymbol{s}}_i^\top\right)\boldsymbol{h}^i_\theta\right) = \boldsymbol{r}^i_t + \frac{\Delta t}{m_i}\boldsymbol{b}^i_\theta \tag{51}$$

Let $\boldsymbol{r}^i_{\mathcal{B}} \in \operatorname{supp}(\pi_{\mathcal{B}})$ be a target point chosen on the ascent ray of $\hat{\boldsymbol{s}}_i$ given by

$$\boldsymbol{r}^i_{\mathcal{B}} \in \left\{\boldsymbol{r}^i_t + \lambda\,\hat{\boldsymbol{s}}_i : \ \lambda \geq 0\right\} \cap \operatorname{supp}(\pi_{\mathcal{B}}) \tag{52}$$

We denote displacement to this target as $\boldsymbol{d}_i := \boldsymbol{r}^i_{\mathcal{B}} - \boldsymbol{r}^i_t = \rho_i\,\hat{\boldsymbol{d}}_i$ with $\hat{\boldsymbol{d}}_i := \frac{\boldsymbol{d}_i}{\|\boldsymbol{d}_i\|}$ and $\rho_i := \|\boldsymbol{d}_i\| \geq 0$. By construction of the ray, $\hat{\boldsymbol{d}}_i = \hat{\boldsymbol{s}}_i$ (i.e., $\boldsymbol{d}_i$ is parallel with $\hat{\boldsymbol{s}}_i$). After a single step, the squared distance is given by

$$\begin{aligned}\|\boldsymbol{r}^i_{\mathcal{B}} - \boldsymbol{r}^i_{t+\Delta t}\|^2 &= \left\|\boldsymbol{r}^i_{\mathcal{B}} - \left(\boldsymbol{r}^i_t + \frac{\Delta t}{m_i}\boldsymbol{b}^i_\theta\right)\right\|^2 \\ &= \left\|\boldsymbol{d}_i - \frac{\Delta t}{m_i}\boldsymbol{b}^i_\theta\right\|^2 \\ &= \|\boldsymbol{d}_i\|^2 - \frac{2\Delta t}{m_i}\langle\boldsymbol{d}_i, \boldsymbol{b}^i_\theta\rangle + \frac{\Delta t^2}{m_i^2}\|\boldsymbol{b}^i_\theta\|^2 \end{aligned} \tag{53}$$

Using the decomposition of $\boldsymbol{b}^i_\theta$ and the fact that $\boldsymbol{d}_i \parallel \hat{\boldsymbol{s}}_i$ and $(\boldsymbol{I} - \hat{\boldsymbol{s}}_i\hat{\boldsymbol{s}}_i^\top)\boldsymbol{h}^i_\theta \perp \hat{\boldsymbol{s}}_i$, we obtain

$$\langle\boldsymbol{d}_i, \boldsymbol{b}^i_\theta\rangle = \alpha^i_\theta\,\langle\boldsymbol{d}_i, \hat{\boldsymbol{s}}_i\rangle + \langle\boldsymbol{d}_i, \underbrace{(\boldsymbol{I} - \hat{\boldsymbol{s}}_i\hat{\boldsymbol{s}}_i^\top)\boldsymbol{h}^i_\theta}_{\perp\,\hat{\boldsymbol{s}}_i}\rangle = \alpha^i_\theta\,\langle\rho_i\hat{\boldsymbol{s}}_i, \hat{\boldsymbol{s}}_i\rangle = \alpha^i_\theta\rho_i \geq 0 \tag{54}$$

Thus, the first-order term in (53) always decreases (or preserves) the distance, and the orthogonal correction cannot increase it at first order because it is orthogonal to $\boldsymbol{d}^i_t$. When $\Delta t \to 0$, the second-order term becomes negligible and the first-order term dominates. We determine the exact threshold for Δt that guarantees a non-increasing distance to be

$$\frac{2\Delta t}{m_i}\alpha^i_\theta\rho_i \geq \frac{\Delta t^2}{m_i^2}\|\boldsymbol{b}^i_\theta\|^2 \implies \Delta t \leq \frac{2m_i\alpha^i_\theta\rho_i}{\|\boldsymbol{b}^i_\theta\|^2} \tag{55}$$

under which the decrease due to the first-order term dominates the quadratic increase, yielding

$$\|\boldsymbol{r}^i_{t+\Delta t} - \boldsymbol{r}^i_{\mathcal{B}}\| \leq \|\boldsymbol{r}^i_t - \boldsymbol{r}^i_{\mathcal{B}}\| \tag{56}$$

In the continuous-time limit as $\Delta t \to 0$, the non-increasing guarantee follows immediately from (54):

$$\frac{\partial}{\partial t}\|\boldsymbol{r}^i_{\mathcal{B}} - \boldsymbol{r}^i_t\|^2 = \frac{\partial}{\partial t}\|\boldsymbol{d}_i\|^2 = -\frac{2}{m_i}\langle \boldsymbol{d}^i_t, \boldsymbol{b}^i_\theta\rangle = -\frac{2}{m_i}\alpha^i_\theta \rho_i \leq 0 \tag{57}$$

Summing over $i = 1, \ldots, n$ (or equivalently using the concatenated coordinates), we conclude that there exists a target configuration $\boldsymbol{R}_{\mathcal{B}} \in \pi_{\mathcal{B}}$ such that the distance to $\boldsymbol{R}_{\mathcal{B}}$ is *non-increasing* under the bias force update, which proves the claim. □

C.3 Proof of Proposition 4.2

Proposition 4.2 (Convexity and Uniqueness of Cross-Entropy Objective)**.** *The cross-entropy objective $\mathcal{L}_{CE}$ is convex in $\mathbb{P}^{b_\theta}$ and there exists a unique minimizer $\boldsymbol{b}^\star$ that is the solution to the EntangledSOC problem in Proposition 3.1.*

Proof. First, we show that the cross-entropy objective is convex in the path measure $\mathbb{P}^{b_\theta}$. The functional $\mathbb{P}^{b_\theta} \mapsto D_{\text{KL}}(\mathbb{P}^\star\|\mathbb{P}^{b_\theta})$ is convex with respect to its second argument, since $x \mapsto -\log x$ is convex. Since $\mathcal{L}_{\text{CE}}(\mathbb{P}^\star, \mathbb{P}^{b_\theta}) := D_{\text{KL}}(\mathbb{P}^\star\|\mathbb{P}^{b_\theta})$ which is strictly convex in $\mathbb{P}^{b_\theta}$, minimizing $\mathcal{L}_{\text{CE}}$ yields a unique minimizer $\mathbb{P}^{b^\star} \equiv \mathbb{P}^\star$. From Proposition 3.1, we have that the unique minimizer of $\mathcal{L}_{\text{CE}}$ is exactly the solution to the EntangledSB problem. While this does not necessarily imply convexity in the neural network parameters θ, it yields a more favorable optimization objective. □

C.4 Proof of Proposition 4.3

Proposition 4.3 (Equivalence of Variational and Path Integral Objectives)**.** *The cross-entropy objective can be expressed in path-integral form as*

$$\mathcal{L}_{CE}(\theta) = \mathbb{E}_{\mathbb{P}^v}\left[w^\star(\boldsymbol{X}_{0:T})\mathcal{F}_{\boldsymbol{b}_\theta,\boldsymbol{v}}(\boldsymbol{X}_{0:T})\right] \tag{14}$$

$$\begin{cases} w^\star(\boldsymbol{X}_{0:T}) = \frac{d\mathbb{P}^\star}{d\mathbb{P}^v}(\boldsymbol{X}_{0:T}) = \frac{e^{r(\boldsymbol{X}_T)}}{Z}\frac{d\mathbb{P}^0}{d\mathbb{P}^v}(\boldsymbol{X}_{0:T}) \\ \mathcal{F}_{\boldsymbol{b}_\theta,\boldsymbol{v}}(\boldsymbol{X}_{0:T}) = \frac{1}{2}\int_0^T\|\boldsymbol{b}_\theta(\boldsymbol{R}_t,\boldsymbol{V}_t)\|^2 - \int_0^T(\boldsymbol{b}_\theta^\top\boldsymbol{v})(\boldsymbol{R}_t,\boldsymbol{V}_t)dt - \int_0^T\boldsymbol{b}_\theta(\boldsymbol{R}_t,\boldsymbol{V}_t)^\top d\boldsymbol{W}_t \end{cases} \tag{15}$$

where we define the reference measure $\boldsymbol{v} = \bar{\boldsymbol{b}} := \texttt{stopgrad}(\boldsymbol{b}_\theta)$ is the off-policy control drift from the previous iteration.

From the cross-entropy objective, we have

$$\mathcal{L}_{\text{CE}}(\mathbb{P}^\star, \mathbb{P}^{b_\theta}) := D_{\text{KL}}(\mathbb{P}^\star\|\mathbb{P}^{b_\theta}) = \mathbb{E}_{\mathbb{P}^\star}\left[\log\frac{d\mathbb{P}^\star}{d\mathbb{P}^{b_\theta}}\right] = \mathbb{E}_{\mathbb{P}^{\bar{b}}}\left[\frac{d\mathbb{P}^\star}{d\mathbb{P}^{\bar{b}}}\log\frac{d\mathbb{P}^\star}{d\mathbb{P}^{b_\theta}}\right] \tag{58}$$

Setting $w^\star := \frac{d\mathbb{P}^\star}{d\mathbb{P}^{\bar{b}}}$, we can derive

$$w^\star(\boldsymbol{X}_{0:T}) = \frac{d\mathbb{P}^\star}{d\mathbb{P}^{\bar{b}}}(\boldsymbol{X}_{0:T}) = \frac{d\mathbb{P}^\star}{d\mathbb{P}^0}\frac{d\mathbb{P}^0}{d\mathbb{P}^{\bar{b}}}(\boldsymbol{X}_{0:T}) \tag{59}$$

From Lemma C.1, we have

$$w^\star(\boldsymbol{X}_{0:T}) = \frac{e^{r(\boldsymbol{X}_T)}}{Z}\frac{d\mathbb{P}^0}{d\mathbb{P}^{\bar{b}}}(\boldsymbol{X}_{0:T}) \tag{60}$$

which is our definition for $w^\star$. Now, expanding $\log\frac{d\mathbb{P}^\star}{d\mathbb{P}^{b_\theta}}$, we have

$$\begin{aligned} \log\frac{d\mathbb{P}^\star}{d\mathbb{P}^{b_\theta}} &= \log\frac{d\mathbb{P}^\star}{d\mathbb{P}^0} + \log\frac{d\mathbb{P}^0}{d\mathbb{P}^{b_\theta}} \\ &= \log\frac{e^{r(\boldsymbol{X}_T)}}{Z} - \log\frac{d\mathbb{P}^{b_\theta}}{d\mathbb{P}^0} \\ &= r(\boldsymbol{X}_T) - \log Z - \log\frac{d\mathbb{P}^{b_\theta}}{d\mathbb{P}^0} \end{aligned} \tag{61}$$

Applying Girsanov's theorem from Lemma B.1, we expand the second term $\log \frac{d\mathbb{P}^{b_\theta}}{d\mathbb{P}^0}$ as

$$\log \frac{d\mathbb{P}^{b_\theta}}{d\mathbb{P}^0}(\boldsymbol{X}_{0:T}) = \int_0^T (\boldsymbol{b}_\theta(\boldsymbol{X}_t)^\top \boldsymbol{\Sigma}^{-1}) d\boldsymbol{X}_t - \int_0^T (\boldsymbol{\Sigma}^{-1}\boldsymbol{f}(\boldsymbol{X}_t))^\top \boldsymbol{b}_\theta(\boldsymbol{X}_t)dt - \frac{1}{2}\int_0^T \|\boldsymbol{b}_\theta(\boldsymbol{X}_t)\|^2 dt$$

Given that $d\boldsymbol{X}_t$ evolves via the SDE

$$d\boldsymbol{X}_t = (\boldsymbol{f}(\boldsymbol{X}_t) + \boldsymbol{\Sigma}\bar{\boldsymbol{b}}(\boldsymbol{X}_t))dt + \boldsymbol{\Sigma} d\boldsymbol{W}_t \tag{62}$$

Applying $(\boldsymbol{b}_\theta^\top \boldsymbol{\Sigma}^{-1})$ to both sides of the equation, we have

$$\begin{aligned}(\boldsymbol{b}_\theta^\top \boldsymbol{\Sigma}^{-1}) d\boldsymbol{X}_t &= (\boldsymbol{b}_\theta^\top \boldsymbol{\Sigma}^{-1})(\boldsymbol{f}(\boldsymbol{X}_t) + \boldsymbol{\Sigma}\bar{\boldsymbol{b}}(\boldsymbol{X}_t))dt + (\boldsymbol{b}_\theta^\top \boldsymbol{\Sigma}^{-1})\boldsymbol{\Sigma} d\boldsymbol{W}_t \\ &= \boldsymbol{b}_\theta^\top (\boldsymbol{\Sigma}^{-1}\boldsymbol{f})(\boldsymbol{X}_t)dt + (\boldsymbol{b}_\theta^\top \boldsymbol{\Sigma}^{-1}\boldsymbol{\Sigma}\bar{\boldsymbol{b}})(\boldsymbol{X}_t)dt + (\boldsymbol{b}_\theta^\top \boldsymbol{\Sigma}^{-1}\boldsymbol{\Sigma})d\boldsymbol{W}_t \\ &= (\boldsymbol{\Sigma}^{-1}\boldsymbol{f}(\boldsymbol{X}_t))^\top \boldsymbol{b}_\theta(\boldsymbol{X}_t)dt + (\boldsymbol{b}_\theta^\top \bar{\boldsymbol{b}})(\boldsymbol{X}_t)dt + \boldsymbol{b}_\theta^\top d\boldsymbol{W}_t \end{aligned} \tag{63}$$

Substituting this into (62), we get

$$\begin{aligned}\log \frac{d\mathbb{P}^{b_\theta}}{d\mathbb{P}^0}(\boldsymbol{X}_{0:T}) &= \int_0^T (\boldsymbol{b}_\theta(\boldsymbol{X}_t)^\top \boldsymbol{\Sigma}^{-1}) d\boldsymbol{X}_t - \int_0^T (\boldsymbol{\Sigma}^{-1}\boldsymbol{f}(\boldsymbol{X}_t))^\top \boldsymbol{b}_\theta(\boldsymbol{X}_t)dt - \frac{1}{2}\int_0^T \|\boldsymbol{b}_\theta(\boldsymbol{X}_t)\|^2 dt \\ &= \int_0^T \left((\boldsymbol{\Sigma}^{-1}\boldsymbol{f}(\boldsymbol{X}_t))^\top \boldsymbol{b}_\theta(\boldsymbol{X}_t)dt + (\boldsymbol{b}_\theta^\top \bar{\boldsymbol{b}})(\boldsymbol{X}_t)dt + \boldsymbol{b}_\theta^\top d\boldsymbol{W}_t\right) \\ &\quad - \int_0^T (\boldsymbol{\Sigma}^{-1}\boldsymbol{f}(\boldsymbol{X}_t))^\top \boldsymbol{b}_\theta(\boldsymbol{X}_t)dt - \frac{1}{2}\int_0^T \|\boldsymbol{b}_\theta(\boldsymbol{X}_t)\|^2 dt \\ &= \int_0^T (\boldsymbol{\Sigma}^{-1}\boldsymbol{f}(\boldsymbol{X}_t))^\top \boldsymbol{b}_\theta(\boldsymbol{X}_t)dt + \int_0^T (\boldsymbol{b}_\theta^\top \bar{\boldsymbol{b}})(\boldsymbol{X}_t)dt + \int_0^T \boldsymbol{b}_\theta^\top d\boldsymbol{W}_t \\ &\quad - \int_0^T (\boldsymbol{\Sigma}^{-1}\boldsymbol{f}(\boldsymbol{X}_t))^\top \boldsymbol{b}_\theta(\boldsymbol{X}_t)dt - \frac{1}{2}\int_0^T \|\boldsymbol{b}_\theta(\boldsymbol{X}_t)\|^2 dt \\ &= \int_0^T (\boldsymbol{b}_\theta^\top \bar{\boldsymbol{b}})(\boldsymbol{X}_t)dt + \int_0^T \boldsymbol{b}_\theta^\top d\boldsymbol{W}_t - \frac{1}{2}\int_0^T \|\boldsymbol{b}_\theta(\boldsymbol{X}_t)\|^2 dt \end{aligned} \tag{64}$$

Putting everything together into the expression for the cross-entropy objective, we get

$$\begin{aligned}&\mathcal{L}_{\text{CE}}(\boldsymbol{b}_\theta) = \mathbb{E}_{\mathbb{P}^v}\left[w^\star(\boldsymbol{X}_{0:T})\left(r(\boldsymbol{X}_T) - \log Z - \log \frac{d\mathbb{P}^{b_\theta}}{d\mathbb{P}^0}(\boldsymbol{X}_{0:T})\right)\right] \\ &= \mathbb{E}_{\mathbb{P}^v}\left[w^\star(\boldsymbol{X}_{0:T})\left(r(\boldsymbol{X}_T) - \log Z - \left[\int_0^T (\boldsymbol{b}_\theta^\top \bar{\boldsymbol{b}})(\boldsymbol{X}_t)dt + \int_0^T \boldsymbol{b}_\theta^\top d\boldsymbol{W}_t - \frac{1}{2}\int_0^T \|\boldsymbol{b}_\theta(\boldsymbol{X}_t)\|^2 dt\right]\right)\right] \\ &= \mathbb{E}_{\mathbb{P}^v}\left[w^\star(\boldsymbol{X}_{0:T})\,(r(\boldsymbol{X}_T) - \log Z) - w^\star(\boldsymbol{X}_{0:T})\left(\int_0^T (\boldsymbol{b}_\theta^\top \bar{\boldsymbol{b}})(\boldsymbol{X}_t)dt + \int_0^T \boldsymbol{b}_\theta^\top d\boldsymbol{W}_t - \frac{1}{2}\int_0^T \|\boldsymbol{b}_\theta(\boldsymbol{X}_t)\|^2 dt\right)\right] \\ &= \mathbb{E}_{\mathbb{P}^v}\Bigg[\underbrace{w^\star(\boldsymbol{X}_{0:T})\,(r(\boldsymbol{X}_T) - \log Z)}_{\text{independent of }\theta} + w^\star(\boldsymbol{X}_{0:T})\underbrace{\left(\frac{1}{2}\int_0^T \|\boldsymbol{b}_\theta(\boldsymbol{X}_t)\|^2 dt - \int_0^T (\boldsymbol{b}_\theta^\top \bar{\boldsymbol{b}})(\boldsymbol{X}_t)dt - \int_0^T \boldsymbol{b}_\theta^\top d\boldsymbol{W}_t\right)}_{:=\mathcal{F}_{\boldsymbol{b}_\theta,\bar{\boldsymbol{b}}}(\boldsymbol{X}_{0:T})}\Bigg]\end{aligned}$$

Since $w^\star(\boldsymbol{X}_{0:T})\,(r(\boldsymbol{X}_T) - \log Z)$ is independent of θ, we can drop it and write the cross-entropy objective as

$$\mathcal{L}_{\text{CE}}(\theta) = \mathbb{E}_{\mathbb{P}^{\bar{b}}}\left[w^\star(\boldsymbol{X}_{0:T})\mathcal{F}_{\boldsymbol{b}_\theta,\bar{\boldsymbol{b}}}(\boldsymbol{X}_{0:T})\right] \tag{65}$$

where we define $\bar{\boldsymbol{b}} := \texttt{stopgrad}(\boldsymbol{b}_\theta)$. □

C.5 Proof of Proposition 4.4

Proposition 4.4 (Discretized Cross-Entropy). *Given the discretized $\hat{\mathcal{F}}_{\boldsymbol{b}_\theta,\bar{\boldsymbol{b}}}(\boldsymbol{X}_{0:K})$ in (16), we can derive a simplified loss function as*

$$\hat{\mathcal{L}}_{CE}(\theta) = \mathbb{E}_{\boldsymbol{X}_{0:K}\sim\mathbb{P}^{\bar{b}}}\Bigg[\underbrace{\frac{d\mathbb{P}^\star}{d\mathbb{P}^{\bar{b}}}(\boldsymbol{X}_{0:K})}_{w^\star(\boldsymbol{X}_{0:K})}\log\left(\frac{p^0(\boldsymbol{X}_{0:K})\exp(r(\boldsymbol{X}_K))}{p^{b_\theta}(\boldsymbol{X}_{0:K})}\right)\Bigg] \tag{17}$$

where $w^\star(\boldsymbol{X}_{0:K})$ is the importance weight of the discrete time trajectory $\boldsymbol{X}_{0:K}$.

We will show that the discretized expression for $\hat{\mathcal{F}}_{\boldsymbol{b}_\theta,\hat{\boldsymbol{b}}}(\boldsymbol{X}_{0:K})$ can be written as

$$\hat{\mathcal{F}}_{\boldsymbol{b}_\theta,\boldsymbol{v}}(\boldsymbol{X}_{0:K}) = \log\left(\frac{p^\star(\boldsymbol{X}_{0:K})}{p^{b_\theta}(\boldsymbol{X}_{0:K})}\right) = \log\left(\frac{p^0(\boldsymbol{X}_{0:K})\exp(r(\boldsymbol{X}_K))}{p^{b_\theta}(\boldsymbol{X}_{0:K})}\right) \tag{66}$$

First, we expand the expression as

$$\begin{aligned}\log\left(\frac{p^0(\boldsymbol{X}_{0:K})\exp(r(\boldsymbol{X}_K))}{p^{b_\theta}(\boldsymbol{X}_{0:K})}\right) &= \log p^0(\boldsymbol{X}_{0:K}) + r(\boldsymbol{X}_K) - \log p^{b_\theta}(\boldsymbol{X}_{0:K}) \\ &= \sum_{k=0}^{K-1}\left[\log p^\star(\boldsymbol{X}_{k+1}|\boldsymbol{X}_k) - \log p^{b_\theta}(\boldsymbol{X}_{k+1}|\boldsymbol{X}_k)\right] + r(\boldsymbol{X}_K) \qquad (67)\end{aligned}$$

The distribution of the position $\boldsymbol{R}_{k+1}$ under the uncontrolled dynamics is a Gaussian with mean $\boldsymbol{R}_k$ and co-variance $\boldsymbol{\Sigma}^\top\boldsymbol{\Sigma}\Delta t$. The log-density becomes

$$\begin{aligned}\log p^0(\boldsymbol{X}_{k+1}|\boldsymbol{X}_k) &= \log\mathcal{N}\left(\boldsymbol{X}_{k+1}\middle|\boldsymbol{X}_k, \boldsymbol{\Sigma}^\top\boldsymbol{\Sigma}\Delta t\right) \\ &= -\frac{1}{2}(\boldsymbol{X}_{k+1}-\boldsymbol{X}_k)^\top(\boldsymbol{\Sigma}^\top\boldsymbol{\Sigma}\Delta t)^{-1}(\boldsymbol{X}_{k+1}-\boldsymbol{X}_k) \qquad (68)\end{aligned}$$

Similarly, the log-density under the bias force $\boldsymbol{b}_\theta$ is given by

$$\begin{aligned}\log p^{b_\theta}(\boldsymbol{X}_{k+1}|\boldsymbol{X}_k) &= \log\mathcal{N}\left(\boldsymbol{X}_{k+1}\middle|\boldsymbol{X}_k + \boldsymbol{\Sigma}\boldsymbol{b}_\theta(\boldsymbol{X}_k)\Delta t, \boldsymbol{\Sigma}^\top\boldsymbol{\Sigma}\Delta t\right) \\ &= -\frac{1}{2}(\boldsymbol{X}_{k+1}-\boldsymbol{X}_k-\boldsymbol{\Sigma}\boldsymbol{b}_\theta(\boldsymbol{X}_k)\Delta t)^\top(\boldsymbol{\Sigma}^\top\boldsymbol{\Sigma}\Delta t)^{-1}(\boldsymbol{X}_{k+1}-\boldsymbol{X}_k-\boldsymbol{\Sigma}\boldsymbol{b}_\theta(\boldsymbol{X}_k)\Delta t) \qquad (69)\end{aligned}$$

Now, we write the increment from time k to $k+1$ as

$$\begin{aligned}\boldsymbol{X}_{k+1} &= \boldsymbol{X}_k + \boldsymbol{\Sigma}\bar{\boldsymbol{b}}(\boldsymbol{X}_k)\Delta t + \boldsymbol{\Sigma}\Delta\boldsymbol{W}_k \\ \boldsymbol{X}_{k+1} - \boldsymbol{X}_k &= \boldsymbol{\Sigma}\bar{\boldsymbol{b}}(\boldsymbol{X}_k)\Delta t + \boldsymbol{\Sigma}\Delta\boldsymbol{W}_k \qquad (70)\end{aligned}$$

and substitute into (68) to get

$$\begin{aligned}&\log p^0(\boldsymbol{X}_{k+1}|\boldsymbol{X}_k) - \log p^{b_\theta}(\boldsymbol{X}_{k+1}|\boldsymbol{X}_k) \\ &= -\frac{1}{2}\left(\boldsymbol{\Sigma}\bar{\boldsymbol{b}}(\boldsymbol{X}_k)\Delta t + \boldsymbol{\Sigma}\Delta\boldsymbol{W}_k\right)^\top\left(\boldsymbol{\Sigma}^\top\boldsymbol{\Sigma}\Delta t\right)^{-1}\left(\boldsymbol{\Sigma}\bar{\boldsymbol{b}}(\boldsymbol{X}_k)\Delta t + \boldsymbol{\Sigma}\Delta\boldsymbol{W}_k\right) \\ &+ \frac{1}{2}\left(\boldsymbol{\Sigma}\bar{\boldsymbol{b}}(\boldsymbol{X}_k)\Delta t + \boldsymbol{\Sigma}\Delta\boldsymbol{W}_k - \boldsymbol{\Sigma}\boldsymbol{b}_\theta(\boldsymbol{X}_k)\Delta t\right)^\top\left(\boldsymbol{\Sigma}^\top\boldsymbol{\Sigma}\Delta t\right)^{-1}\left(\boldsymbol{\Sigma}\bar{\boldsymbol{b}}(\boldsymbol{X}_k)\Delta t + \boldsymbol{\Sigma}\Delta\boldsymbol{W}_k - \boldsymbol{\Sigma}\boldsymbol{b}_\theta(\boldsymbol{X}_k)\Delta t\right)\end{aligned}$$

Given that $\left(\boldsymbol{\Sigma}^\top\boldsymbol{\Sigma}\Delta t\right)^{-1} = \frac{1}{\Delta t}(\boldsymbol{\Sigma}^\top\boldsymbol{\Sigma})^{-1} = \frac{1}{\Delta t}(\boldsymbol{\Sigma}^{-1}\boldsymbol{\Sigma}^{-\top})$, and rewriting $\boldsymbol{a} = \bar{\boldsymbol{b}}\Delta t + \Delta\boldsymbol{W}_k - \boldsymbol{b}_\theta\Delta t$ and $\bar{\boldsymbol{a}} = \bar{\boldsymbol{b}}\Delta t + \Delta\boldsymbol{W}_k$, we have

$$\begin{aligned}&\log p^0(\boldsymbol{X}_{k+1}|\boldsymbol{X}_k) - \log p^{b_\theta}(\boldsymbol{X}_{k+1}|\boldsymbol{X}_k) \\ &= \frac{1}{2\Delta t}\left[-(\boldsymbol{\Sigma}\bar{\boldsymbol{a}})^\top(\boldsymbol{\Sigma}^{-1}\boldsymbol{\Sigma}^{-\top})(\boldsymbol{\Sigma}\bar{\boldsymbol{a}}) + (\boldsymbol{\Sigma}\boldsymbol{a})^\top(\boldsymbol{\Sigma}^{-1}\boldsymbol{\Sigma}^{-\top})(\boldsymbol{\Sigma}\boldsymbol{a})\right] \\ &= \frac{1}{2\Delta t}\left[-\bar{\boldsymbol{a}}^\top\left(\boldsymbol{\Sigma}^\top\boldsymbol{\Sigma}^{-1}\boldsymbol{\Sigma}^{-\top}\boldsymbol{\Sigma}\right)\bar{\boldsymbol{a}} + \boldsymbol{a}^\top\left(\boldsymbol{\Sigma}^\top\boldsymbol{\Sigma}^{-1}\boldsymbol{\Sigma}^{-\top}\boldsymbol{\Sigma}\right)\boldsymbol{a}\right] \\ &= \frac{1}{2\Delta t}\left[-\|\bar{\boldsymbol{a}}\|^2 + \|\boldsymbol{a}\|^2\right] \\ &= \frac{1}{2\Delta t}\left[-\|\bar{\boldsymbol{b}}\Delta t + \Delta\boldsymbol{W}_k\|^2 + \|\bar{\boldsymbol{b}}\Delta t + \Delta\boldsymbol{W}_k - \boldsymbol{b}_\theta\Delta t\|^2\right] \\ &= \frac{1}{2\Delta t}\left[-\|\bar{\boldsymbol{b}}\Delta t + \Delta\boldsymbol{W}_k\|^2 + \|\bar{\boldsymbol{b}}\Delta t + \Delta\boldsymbol{W}_k\|^2 - 2(\boldsymbol{b}_\theta\Delta t)\left(\bar{\boldsymbol{b}}\Delta t + \Delta\boldsymbol{W}_k\right) + \|\boldsymbol{b}_\theta\Delta t\|^2\right] \\ &= \frac{1}{2\Delta t}\left[-2(\boldsymbol{b}_\theta\cdot\bar{\boldsymbol{b}})(\Delta t)^2 - 2(\boldsymbol{b}_\theta\cdot\Delta\boldsymbol{W}_k)\Delta t + \|\boldsymbol{b}_\theta\|^2(\Delta t)^2\right] \\ &= -(\boldsymbol{b}_\theta\cdot\bar{\boldsymbol{b}})\Delta t - (\boldsymbol{b}_\theta\cdot\Delta\boldsymbol{W}_k) + \frac{1}{2}\|\boldsymbol{b}_\theta\|^2\Delta t \qquad (71)\end{aligned}$$

Summing over all K steps and adding $r(\boldsymbol{X}_K)$, we have

$$\begin{aligned}&\sum_{k=0}^{K-1}\left[\log p^{b_\theta}(\boldsymbol{X}_{k+1}|\boldsymbol{X}_k) - \log p^0(\boldsymbol{X}_{k+1}|\boldsymbol{X}_k)\right] + r(\boldsymbol{X}_K) \\ &= \underbrace{\frac{1}{2}\sum_{k=0}^{K-1}\|\boldsymbol{b}_\theta(\boldsymbol{X}_k)\|^2\Delta t - \sum_{k=0}^{K-1}(\boldsymbol{b}_\theta\cdot\bar{\boldsymbol{b}})(\boldsymbol{X}_k)\Delta t - \sum_{k=0}^{K-1}\boldsymbol{b}_\theta(\boldsymbol{X}_k)\cdot\Delta\boldsymbol{W}_k + r(\boldsymbol{X}_K)}_{:=\hat{\mathcal{F}}_{\boldsymbol{b}_\theta,\bar{\boldsymbol{b}}}(\boldsymbol{X}_{0:K})} \qquad (72)\end{aligned}$$

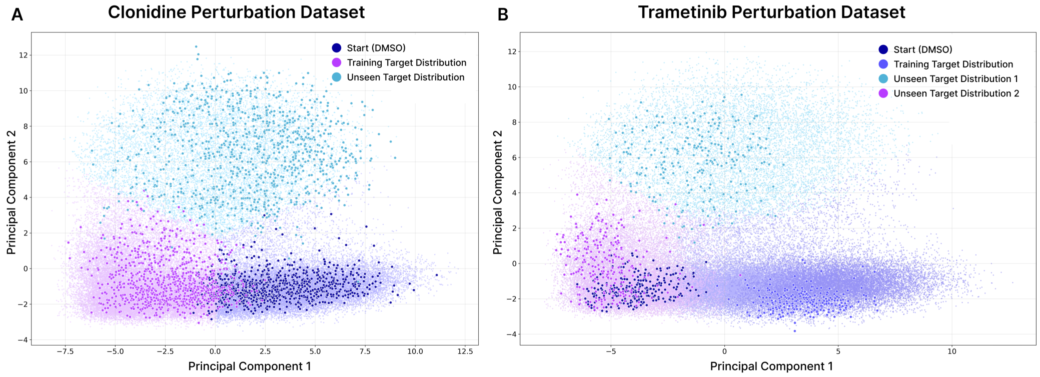

Figure 5: **Training and held-out cell clusters for the cell perturbation experiment.** All cells are plotted with the top 2 PCs. **(A)** Clonidine ($5\mu L$) perturbation data containing the initial DMSO-treated control cells (dark blue), perturbed cluster for training (magenta), and held-out validation cluster (turquoise). **(B)** Trametinib ($5\mu L$) perturbation data containing the initial DMSO-treated control cells (navy blue), the perturbed cluster for training (purple), and two held-out perturbed clusters (magenta and turquoise).

Therefore, we can write $\hat{\mathcal{F}}_{\boldsymbol{b}_\theta,\bar{\boldsymbol{b}}}(\boldsymbol{X}_{0:K})$ equal to

$$\hat{\mathcal{F}}_{\boldsymbol{b}_\theta,\bar{\boldsymbol{b}}}(\boldsymbol{X}_{0:K}) = \sum_{k=0}^{K-1} \log \frac{p^0(\boldsymbol{X}_{k+1}|\boldsymbol{X}_k)}{p^{b_\theta}(\boldsymbol{X}_{k+1}|\boldsymbol{X}_k)} + r(\boldsymbol{X}_K) = \log\left(\frac{p^0(\boldsymbol{X}_{0:K})\exp(r(\boldsymbol{X}_K))}{p^{b_\theta}(\boldsymbol{X}_{0:K})}\right) \tag{73}$$

where $r(\boldsymbol{X}_K) := \log \pi_{\mathcal{B}}(\boldsymbol{X}_K)$. □

D Cell Perturbation Experiment Details

D.1 Experiment Setup

Data Processing For this experiment, we extract the cell perturbation data from the Tahoe-100M dataset consists of 50 cell lines and over 1000 different drug-dose conditions (Zhang et al., 2025a). Specifically, we use data on a single cell line (A-549) under two drug perturbation conditions: Clonidine at $5\mu M$ and Trametinib at $5\mu M$. The initial distribution is defined as the DMSO-treated control cells. Using centroid-based sampling, we obtain balanced training sets of 1033 cells per cluster (Figure 5). We split the cells into training and validation sets with a 0.9/0.1 ratio. All final visualizations are plotted with the first two principal components, but the cell state trajectories are simulated for $d \in \{50, 100, 150\}$ for Clonidine and $d = 50$ for Trametinib.

Table 3: **Training cluster cell counts for perturbation experiments.**

	**Clonidine**		**Trametinib**		
	Cluster 1	Cluster 2	Cluster 1	Cluster 2	Cluster 3
Original Cell Count	1675	1033	1622	686	381

Model Architecture To learn the dependencies across the n particles, we use Transformer blocks to learn dependencies between the positions $\boldsymbol{R}_t \in \mathbb{R}^{n\times d}$ and velocities $\boldsymbol{V}_t \in \mathbb{R}^{n\times d}$ in the system. The input to the model is the concatenated position and velocity vectors $\texttt{Cat}(\boldsymbol{R}_t, \boldsymbol{V}_t) \in \mathbb{R}^{n\times 6}$. The architecture consists of an input projection layer that projects the input to $d_{\text{hidden}} = 256$, a 4-layer Transformer Encoder (`gelu` activation, feedforward dimension $d_{\text{ff}} = 512$, 8 attention heads, and dropout 0.1), and two MLP decoder heads that predict the scaling factor $\alpha_t(\boldsymbol{R}_t, \boldsymbol{V}_t)$ and correction vector $\boldsymbol{h}_t^i(\boldsymbol{R}_t, \boldsymbol{V}_t)$ per atom. To ensure positive scaling, the `softplus` activation is applied to the output of the scalar MLP head. The correction vector is projected onto the orthogonal plane of the vector pointing in the direction of the target $\hat{\boldsymbol{s}}_i$, and the output bias force is the sum of the scaled directional unit vector $\alpha_\theta^i(\boldsymbol{R}_t, \boldsymbol{V}_t)\hat{\boldsymbol{s}}_i$ and the projected correction vector $(\boldsymbol{I} - \hat{\boldsymbol{s}}_i\hat{\boldsymbol{s}}_i^\top)\boldsymbol{h}_\theta^i(\boldsymbol{R}_t, \boldsymbol{V}_t)$.

Terminal Reward To solve the EntangledSB problem, we defined the terminal reward to be the log-probability under the target density $\pi_{\mathcal{B}}$ given by $r(\boldsymbol{X}_T) = \log \pi_{\mathcal{B}}(\boldsymbol{R}_T)$. We set the target density as the Gaussian centered around $\boldsymbol{R}_{\mathcal{B}}$ with radius σ.

$$\pi_{\mathcal{B}}(\boldsymbol{R}_T) = \frac{1}{(2\pi\sigma^2)^{\frac{d}{2}}} \exp\left(\frac{-\|\boldsymbol{R}_T - \boldsymbol{R}_{\mathcal{B}}\|_2^2}{2\sigma^2}\right) \tag{74}$$

Therefore, to train the bias force to reconstruct the input target state of a cell cluster $\boldsymbol{R}_{\mathcal{B}} \in \mathbb{R}^{n\times d}$, we define $r : \mathcal{X} \to \mathbb{R}$ as:

$$r(\boldsymbol{X}_T) := \frac{-\|\boldsymbol{R}_T - \boldsymbol{R}_{\mathcal{B}}\|_2^2}{2\sigma^2} \tag{75}$$

The specific values for σ are provided in Table 4.

Simulating Trajectories on the Data Manifold To define an energy landscape on the data manifold, we use the RBF metric introduced in Kapuśniak et al. (2024), which is lower in magnitude when $\boldsymbol{x}$ is within the support of the data manifold and larger in magnitude when $\boldsymbol{x}$ moves away from the support of the dataset into sparse regions. First, we define the elements of a function h_j^{RBF} that satisfies $h_j^{\text{RBF}}(\boldsymbol{x}) \approx 1$ on the data manifold as

$$h_j^{\text{RBF}}(\boldsymbol{x}) = \sum_{m=1}^{N_c} \omega_{m,j}(\boldsymbol{x}) \exp\left(-\frac{\lambda_m}{2}\|\boldsymbol{x} - \hat{\boldsymbol{x}}_n\|^2\right) \tag{76}$$

$$\lambda_m = \frac{1}{2}\left(\frac{\kappa}{|C_m|}\sum_{\boldsymbol{x}\in C_m}\|\boldsymbol{x} - \hat{\boldsymbol{x}}_m\|^2\right)^{-2} \tag{77}$$

where $\{\omega_{m,j}\}_{m=1}^{N_c}$ for each cluster and each coordinate are learned given a dataset $\mathcal{D}$ to enforce $h_j^{\text{RBF}}(\boldsymbol{x}) \approx 1$ for all $\boldsymbol{x} \in \mathcal{D}$ with the following loss function

$$\mathcal{L}_{\text{RBF}}(\{\omega_{m,j}\}) = \sum_{\boldsymbol{x}_i \in \mathcal{D}} (1 - h_j^{\text{RBF}}(\boldsymbol{x}_i))^2 \tag{78}$$

Then, we define the potential energy function of the base dynamics as

$$U(\boldsymbol{x}) = -\sum_{j=1}^{d} \log(M_j(\boldsymbol{x}) + \varepsilon), \quad \text{where} \quad M_j(\boldsymbol{x}) = \frac{1}{(h_j^{\text{RBF}}(\boldsymbol{x}) + \varepsilon)^{\alpha}} \tag{79}$$

where $M_j(\boldsymbol{x})$ is small in regions of high data density and large in regions of low data density. The resulting force $-\nabla_{\boldsymbol{x}} U(\boldsymbol{x})$ is used by the simulator as the natural-gradient step.

Hyperparameters We present the hyperparameters used for the cell perturbation modeling experiment in Table 4. For each perturbation, we conducted ablations while keeping all other parameters constant on: **(1)** using the log-variance divergence with the learnable control variate $\mathcal{L}_{\text{LV}}$ described in B.1 instead of the cross-entropy objective $\mathcal{L}_{\text{CE}}$ and **(2)** removing the dependency on velocities as a feature input to the bias force model $\boldsymbol{b}_\theta(\boldsymbol{R}_t)$. The hyperparameters of the Transformer architecture are given in App D.1 and are kept constant across all experiments. All models are trained with the Adam optimizer (Kingma & Ba, 2014) with learning rate $\eta = 0.0001$. Due to the small batch sizes used for training, we leverage the importance weights $w^\star(\boldsymbol{X}_{0:T})$ for categorical sampling from the replay buffer $\mathcal{R}$ as $\boldsymbol{X}_{0:T} \sim \texttt{Cat}(\texttt{softmax}_{\mathcal{R}}(w^\star(\boldsymbol{X}_{0:T})))$ to mimic the effect of the reweighting in the cross-entropy objective $\mathcal{L}_{\text{CE}}$ over a larger batch size.

D.2 Evaluation Metrics

Maximum Mean Discrepency (RBF-MMD) We evaluate reconstruction accuracy of the target distribution and the distribution simulated with EntangledSBM using MMD with the RBF kernel (RBF-MMD) on all d principal components used during training. For Clonidine, we evaluate $d \in \{50, 100, 150\}$ and for Trametinib, we evaluate $d = 50$. Given the simulated endpoints of M

Table 4: **Hyperparameter settings for cell perturbation experiment.** The Clonidine perturbation experiment is split into three columns for each of the three dimensions of principal components (PCs) used $d \in \{50, 100, 150\}$.

Parameter	Clonidine			Trametinib
	50 PCs	100 PCs	150 PCs	50 PCs
number of rollouts N_{rollouts}	100	100	100	100
trains per rollout N_{epochs}	1000	1000	1000	1000
step size Δt	0.01	0.01	0.01	0.01
total time steps T	100	100	100	100
number of samples M	64	64	64	64
number of particles n	16	16	16	16
batch size N_{batch}	64	64	64	64
buffer size $\|\mathcal{R}\|$	1000	1000	1000	1000
radius σ	0.1	0.1	0.1	0.1
friction γ	2.0	2.0	2.0	2.0
learning rate	0.0001	0.0001	0.0001	0.0001
RBF N_c	150	300	300	150
RBF κ	1.5	2.0	3.0	1.5

paths for M cell clusters $\{\boldsymbol{R}_T \in \mathbb{R}^{n\times d}\}_{j=1}^M$ and the target states $\{\boldsymbol{R}_\mathcal{B} \in \mathbb{R}^{n\times d}\}_{\ell=1}^M$, the RBF-MMD is calculated as

$$\text{RBF-MMD} = \frac{1}{M^2}\sum_{j=1}^M\sum_{\ell=1}^M k_{\text{mix}}(\boldsymbol{R}_T^j, \boldsymbol{R}_T^\ell) + \frac{1}{M^2}\sum_{j=1}^M\sum_{\ell=1}^M k_{\text{mix}}(\boldsymbol{R}_\mathcal{B}^j, \boldsymbol{R}_\mathcal{B}^\ell) - \frac{2}{M^2}\sum_{j=1}^M\sum_{\ell=1}^M k_{\text{mix}}(\boldsymbol{R}_T^j, \boldsymbol{R}_\mathcal{B}^\ell) \tag{80}$$

where we define the mixture of RBF kernel functions $k_{\text{mix}}(\cdot,\cdot)$ as

$$k_{\text{mix}}(\boldsymbol{R}, \boldsymbol{R}') = \frac{1}{|\Sigma|}\sum_{\sigma\in\Sigma}\exp\left(-\frac{\|\boldsymbol{R}-\boldsymbol{R}'\|^2}{2\sigma^2}\right) \tag{81}$$

for $\Sigma = \{0.01, 0.1, 1, 10, 100\}$.

1-Wasserstein ($\mathcal{W}_1$) and 2-Wasserstein ($\mathcal{W}_2$) Distances We further compute the $\mathcal{W}_1$ and $\mathcal{W}_2$ distances for the top two PCs of the simulated endpoints of M paths for M cell clusters $\{\boldsymbol{R}_T \in \mathbb{R}^{n\times d}\}_{j=1}^M$ which form the predicted distribution π_T and the *full distribution* that the target states are sampled from $\pi_\mathcal{B}$ since the $\mathcal{W}_1$ and $\mathcal{W}_2$ distances can be calculated for a pair of distributions with different sizes. Concretely, the $\mathcal{W}_1$ and $\mathcal{W}_2$ distances are calculated as

$$\mathcal{W}_1 = \left(\min_{\pi\in\Pi(\pi_T,\pi_\mathcal{B})}\int \|\boldsymbol{R}_T - \boldsymbol{R}_\mathcal{B}\|_2 d\pi(\boldsymbol{R}_T, \boldsymbol{R}_\mathcal{B})\right) \tag{82}$$

$$\mathcal{W}_2 = \left(\min_{\pi\in\Pi(\pi_T,\pi_\mathcal{B})}\int \|\boldsymbol{R}_T - \boldsymbol{R}_\mathcal{B}\|_2^2 d\pi(\boldsymbol{R}_T, \boldsymbol{R}_\mathcal{B})\right)^{1/2} \tag{83}$$

which quantify the minimal effort required to transform the simulated endpoint distribution into the true target distribution, demonstrating the ability of the model to capture the true perturbation dynamics.

E Transition Path Sampling Experiment Details

E.1 Experiment Setup

Model Architecture We use the same model architecture as the cell perturbation experiment described in Sec D, with the addition of Kabsch alignment (Kabsch, 1976). We define the **aligned frame** as the frame of the target coordinates and align the input positions and velocities of the *heavy atoms* (non-hydrogen atoms) to the aligned frame. The model then predicts the optimal bias force in the aligned frame, which we transform back to the original frame of the input positions.

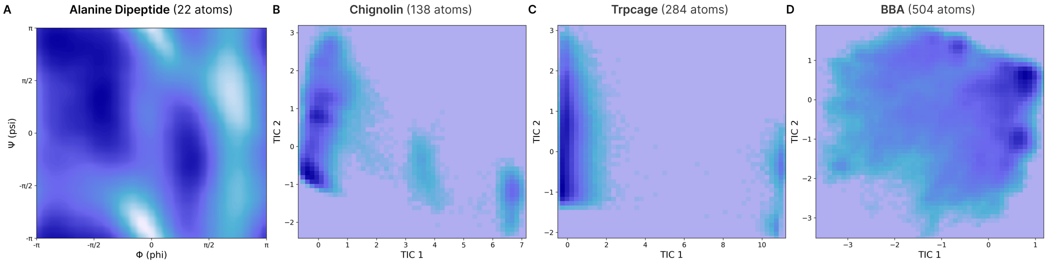

Figure 6: **Energy landscapes for transition path sampling experiments.** Potential energy plotted on dihedral angles (ϕ, ψ) for Alanine Dipeptide and the top two TICA components for fast-folding proteins.

Terminal Reward Following the cell perturbation experiment, given the coordinates of a single target state $\boldsymbol{R}_{\mathcal{B}}$, we define $\pi_{\mathcal{B}}$ as the log-probability under the target Gaussian centered around the coordinates of $\boldsymbol{R}_{\mathcal{B}}$ with radius σ:

$$r(\boldsymbol{X}_T) := \exp\left(\frac{-\|\boldsymbol{R}_T - \boldsymbol{R}_{\mathcal{B}}\|_2^2}{2\sigma^2}\right) \tag{84}$$

The specific values for σ are provided in Table 5.

Molecular Dynamics Setup We closely follow the setup in (Seong et al., 2025). To simulate the position and velocity via the Langevin SDEs, we use the Velocity-Verlet with Velocity Randomization (VVVR) integrator (Sivak et al., 2014) in OpenMM (Eastman et al., 2017) that updates the state with a velocity-Verlet step for the deterministic forces and a stochastic Ornstein-Uhlenbeck step that randomizes velocities. We set the friction parameter to $\gamma = 1\text{ps}^{-1}$ and the time step size to $\Delta t = 1\text{fs}$ for a total time horizon of $T = 1000\text{fs}$ for Alanine Dipeptide and $T = 5000\text{fs}$ for the fast-folding proteins. Following (Seong et al., 2025), we leverage temperature annealing with a starting temperature of $\tau_{\textbf{start}} = 600\text{K}$ and a final temperature of $\tau_{\textbf{end}} = 300\text{K}$ for Alanine Dipeptide and Chignolin and $\tau_{\textbf{end}} = 400\text{K}$ for the remaining fast-folding proteins.

To simulate the unconditional dynamics following the potential energy landscape $U(\boldsymbol{R}_t)$ visualized in Fig. 6 and the corresponding force field $F = -\nabla_{\boldsymbol{R}_t} U(\boldsymbol{R}_t)$, we use the AMBER99SB force field with the ILDN side-chain torsion corrections (`amber99sbildn`) force field (Lindorff-Larsen et al., 2010) for Alanine Dipeptide in a vacuum and the `ff14SBonlysc` forcefield (Maier et al., 2015) paired with the `gbn2` solvation model (Nguyen et al., 2013) for the fast-folding proteins.

Hyperparameters We present the hyperparameters used for the TPS experiment in Table 5. The hyperparameters of the Transformer architecture are given in App D.1 and are kept constant across all experiments. We run the benchmark against TPS-DPS (Seong et al., 2025) with the hyperparameters below to ensure fair comparison. All models are trained with the Adam optimizer (Kingma & Ba, 2014) with learning rate $\eta = 1 \times 10^{-4}$.

E.2 Evaluation Metrics

We follow the evaluations of (Seong et al., 2025; Holdijk et al., 2023) and report three metrics: Root Mean Square Distance (RMSD), Target Hit Percentage (THP), and Energy of Transition State (ETS).

Root Mean Square Distance (RMSD) RMSD (↓) measures the distance between the *heavy atoms* (non-hydrogen atoms) of the final position of the system at time $\boldsymbol{R}_T$ and the target position $\boldsymbol{R}_{\mathcal{B}}$. To align the coordinate frames, we use the Kabsch algorithm (Kabsch, 1976), which determines the optimal rotation and translation such that two pairs of heavy atoms are aligned. The mean and standard deviation across the hits for M paths are reported.

Target Hit Percentage (THP) THP (↑) measures the percentage of the total trajectories where the final position $\boldsymbol{R}_T$ reaches the vicinity of the target state $\boldsymbol{R}_{\mathcal{B}}$. Specifically, we consider a hit when the two backbone dihedral angles (ϕ, ψ) for Alanine Dipeptide or the first two TICA components for the

Table 5: **Hyperparameter settings for transition path sampling experiment.** The setup follows that of Seong et al. (2025) to ensure fair comparison.

Parameter	Task			
	Alanine Dipeptide	Chignolin	Trp-cage	BBA
number of rollouts N_{rollouts}	100	100	100	100
trains per rollout N_{epochs}	1000	1000	1000	1000
step size Δt	$1fs$	$1fs$	$1fs$	$1fs$
total time steps T	1000	5000	5000	5000
number of samples M	64	64	64	64
number of particles n	22	138	284	504
batch size N_{batch}	16	1	1	1
buffer size $\lvert\mathcal{R}\rvert$	1000	100	100	100
starting temperature (Kelvin)	600	600	600	600
ending temperature (Kelvin)	300	300	400	400
radius σ	0.1	0.5	0.5	0.5
friction γ	0.001	0.001	0.001	0.001
learning rate	0.0001	0.0001	0.0001	0.0001

fast-folding proteins (Chignolin, Trp-cage, BBA), denoted $\xi(\boldsymbol{R})$, are within the 0.75-radius sphere around the target defined as $\pi_{\mathcal{B}} = \{\boldsymbol{R} \mid \|\xi(\boldsymbol{R}) - \xi(\boldsymbol{R}_{\mathcal{B}})\| < 0.75\}$. For M total paths, the THP is calculated as

$$\text{THP} = \frac{\sum_{i=1}^{M} \mathbf{1}[\boldsymbol{R}_T^i \in \pi_{\mathcal{B}}]}{M} \tag{85}$$

Energy of Transition State (ETS) ETS (↓) measures the maximum potential energy returned by $U : \mathcal{X} \to \mathbb{R}$ along the discrete transition path $\boldsymbol{R}_{0:K}$ in kJmol^{-1}. It is calculated for the trajectories that reach the vicinity of the target state $\boldsymbol{R}_T \in \pi_{\mathcal{B}}$ and is classified as a hit by the THP metric.

$$\text{ETS}(\boldsymbol{R}_{0:K}) = \max_{k \in \{1,\ldots K\}} U(\boldsymbol{R}_k) \tag{86}$$

The mean and standard deviation across the hits for M paths are reported.

F Comparison to Log-Variance Divergence

In this section, we discuss the intuition behind the differences in cell state trajectories generated with the cross-entropy (CE) objective and log-variance (LV) objective for Clonidine, as shown in Fig. 7 and Table 6, and Trametinib as shown in Fig. 8 and Table 7.

We observe that the log-variance (LV) objective fails to accurately simulate the intermediate dynamics of cells following perturbation and generates nearly straight, abrupt trajectories connecting the initial and terminal populations. The trajectories generated from the LV-trained bias forces ignore the gradual, curved progression of cell states through intermediate data manifolds, in contrast to the trajectories generated from the cross-entropy (CE)-trained bias forces (Fig. 7, 8). While the LV-objective accurately reconstructs the target cell distribution, it fails to reconstruct the intermediate dynamics that arise from nonlinear coupling between position and velocity fields after perturbation (Tables 6, 7).

While the LV objective aims to make the log–likelihood ratio $\log \frac{\mathrm{d}\mathbb{P}^\star}{\mathrm{d}\mathbb{P}^{b_\theta}}(\boldsymbol{X}_{0:T})$ constant *in expectation* to minimize the variance, it is easiest to only maximize the terminal reward $r(\boldsymbol{X}_T)$ rather than intermediate path alignment with $\mathbb{P}^0$. In contrast, the CE objective is defined as the KL-divergence $D_{\text{KL}}(\mathbb{P}^\star \| \mathbb{P}^{b_\theta})$ which expands into a path-integral action term $\frac{1}{2}\int_0^T \|\boldsymbol{b}_\theta(\boldsymbol{R}_t, \boldsymbol{V}_t)\|^2 dt$ using Girsanov's theorem. This explicitly regularizes the whole trajectory, rewarding gradual, smooth transport through intermediate states.

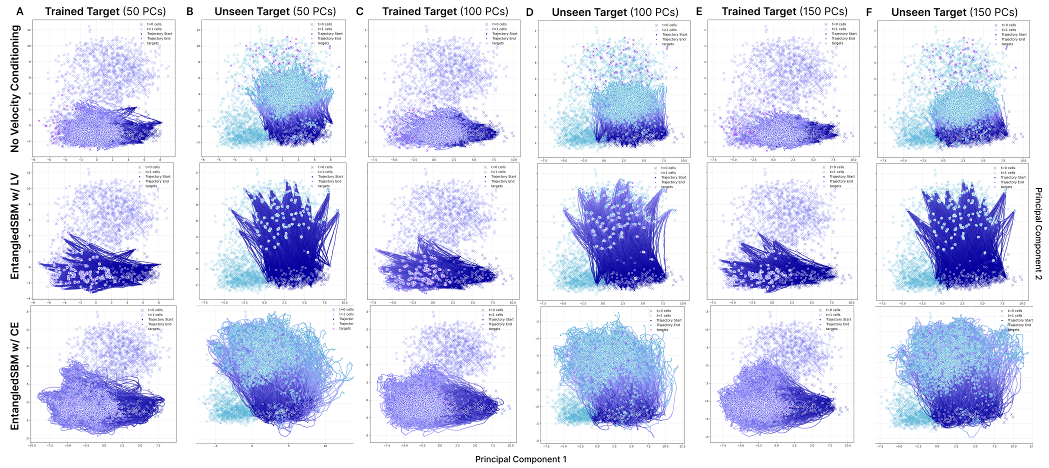

Figure 7: **Full visualization of simulated cell cluster dynamics with EntangledSBM under Clonidine perturbation.** Nearest neighbour cell clusters with $n = 16$ cells are simulated over 100 time steps. The gradient indicates the evolution of timesteps from the initial time $t = 0$ (navy) to the final time $t = T$ (purple or turquoise). 50 PCs simulated to **(A)** trained target distribution and **(B)** unseen target. 100 PCs simulated to **(C)** trained target distribution and **(D)** unseen target. 150 PCs simulated to **(E)** trained target distribution and **(F)** unseen target.

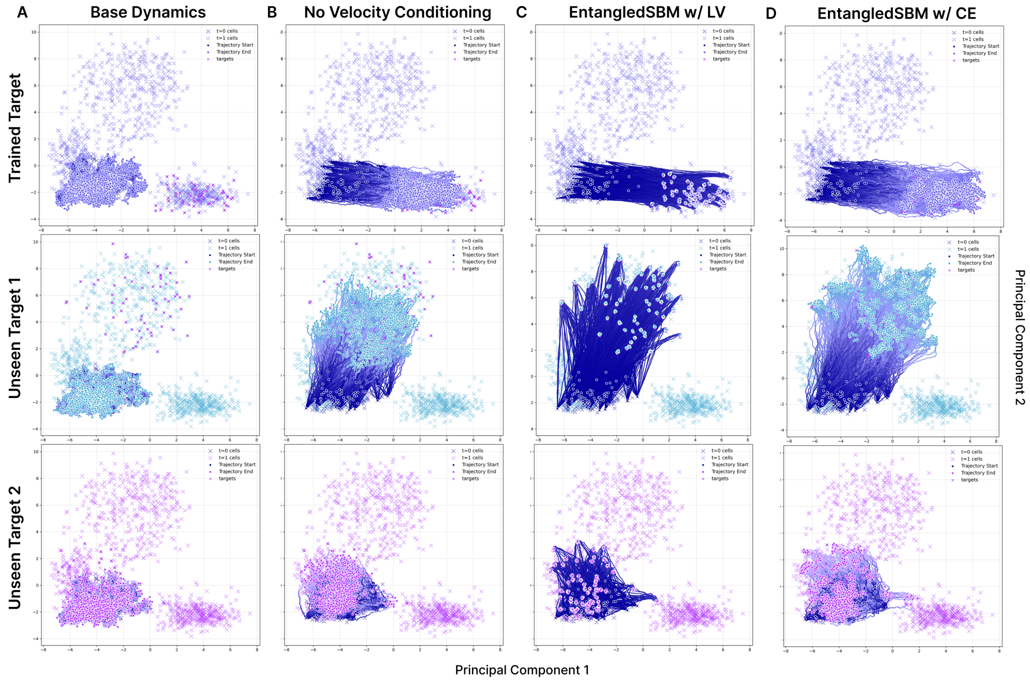

Figure 8: **Full visualization of simulated cell cluster dynamics with EntangledSBM under Trametinib perturbation.** All trajectories generated for $d = 50$ PCs. Nearest neighbour cell clusters with $n = 16$ cells are simulated over 100 time steps. The gradient indicates the evolution of timesteps from the initial time $t = 0$ (navy) to the final time $t = T$ (purple, turquoise, or magenta). **(A)** Base dynamics with no bias force. Dynamics with base and learned bias force trained with **(B)** no velocity conditioning, **(C)** log-variance (LV) objective, and **(D)** cross-entropy objective to the trained target distribution and two unseen target distributions.

Table 6: **Full comparisons for simulating cell cluster dynamics under Clonidine perturbation with EntangledSBM.** We report RBF-MMD for all d PCs with the target cluster and $\mathcal{W}_1$ and $\mathcal{W}_2$ distances of top 2 PCs against the full distribution of the perturbed cells for the seen and unseen populations after 100 simulation steps and cluster size set to $n = 16$. Mean and standard deviation of metrics from 5 independent simulations are reported. Comparisons include the base dynamics, without velocity conditioning, and EntangledSBM with the log-variance (LV) objective instead of cross-entropy (CE) for increasing principal component dimensions $d = \{50, 100, 150\}$.

	**Seen Target Distribution**			**Unseen Target Distribution**		
**Model**	RBF-MMD (↓)	$\mathcal{W}_1$ (↓)	$\mathcal{W}_2$ (↓)	RBF-MMD (↓)	$\mathcal{W}_1$ (↓)	$\mathcal{W}_2$ (↓)
**Base Dynamics** (50 PCs)	$0.677_{\pm 0.001}$	$5.947_{\pm 0.005}$	$6.015_{\pm 0.005}$	$0.784_{\pm 0.001}$	$8.217_{\pm 0.005}$	$8.384_{\pm 0.005}$
**EntangledSBM w/o Velocity Conditioning**						
50 PCs	$0.440_{\pm 0.000}$	$1.741_{\pm 0.003}$	$1.857_{\pm 0.004}$	$0.478_{\pm 0.000}$	$2.907_{\pm 0.006}$	$3.022_{\pm 0.006}$
100 PCs	$0.494_{\pm 0.000}$	$2.315_{\pm 0.004}$	$2.423_{\pm 0.004}$	$0.539_{\pm 0.000}$	$4.110_{\pm 0.004}$	$4.249_{\pm 0.003}$
150 PCs	$0.510_{\pm 0.000}$	$2.497_{\pm 0.006}$	$2.620_{\pm 0.006}$	$0.560_{\pm 0.000}$	$4.573_{\pm 0.006}$	$4.716_{\pm 0.007}$
**EntangledSBM w/ LV**						
50 PCs	$0.294_{\pm 0.000}$	$0.205_{\pm 0.001}$	$0.285_{\pm 0.001}$	$0.290_{\pm 0.000}$	$0.230_{\pm 0.001}$	$0.382_{\pm 0.001}$
100 PCs	$0.330_{\pm 0.000}$	$0.277_{\pm 0.001}$	$0.332_{\pm 0.001}$	$0.346_{\pm 0.000}$	$0.358_{\pm 0.001}$	$0.492_{\pm 0.001}$
150 PCs	$0.323_{\pm 0.000}$	$0.128_{\pm 0.001}$	$0.278_{\pm 0.000}$	$0.327_{\pm 0.000}$	$0.193_{\pm 0.001}$	$0.425_{\pm 0.001}$
**EntangledSBM w/ CE**						
50 PCs	$0.401_{\pm 0.000}$	$0.342_{\pm 0.002}$	$0.400_{\pm 0.001}$	$0.419_{\pm 0.000}$	$0.538_{\pm 0.013}$	$0.705_{\pm 0.030}$
100 PCs	$0.455_{\pm 0.000}$	$0.953_{\pm 0.025}$	$1.015_{\pm 0.025}$	$0.500_{\pm 0.001}$	$0.899_{\pm 0.006}$	$1.055_{\pm 0.008}$
150 PCs	$0.478_{\pm 0.000}$	$0.753_{\pm 0.008}$	$0.826_{\pm 0.007}$	$0.506_{\pm 0.000}$	$0.700_{\pm 0.009}$	$0.811_{\pm 0.011}$

Table 7: **Full comparisons for simulating cell cluster dynamics under Trametinib perturbation with EntangledSBM.** We report RBF-MMD for all $d = 50$ PCs with the target cluster and $\mathcal{W}_1$ and $\mathcal{W}_2$ distances of top 2 PCs against the full distribution of the perturbed cells for the seen and unseen populations after 100 simulation steps and cluster size set to $n = 16$. Mean and standard deviation of metrics from 5 independent simulations are reported. Comparisons include the base dynamics, without velocity conditioning, and EntangledSBM with the log-variance (LV) objective instead of cross-entropy (CE).

	**Seen Target Distribution**			**Unseen Target Distribution 1**			**Unseen Target Distribution 2**		
**Method**	RBF-MMD (↓)	$\mathcal{W}_1$ (↓)	$\mathcal{W}_2$ (↓)	RBF-MMD (↓)	$\mathcal{W}_1$ (↓)	$\mathcal{W}_2$ (↓)	RBF-MMD (↓)	$\mathcal{W}_1$ (↓)	$\mathcal{W}_2$ (↓)
**Base Dynamics**	$0.938_{\pm 0.001}$	$7.637_{\pm 0.005}$	$7.653_{\pm 0.006}$	$0.900_{\pm 0.000}$	$7.766_{\pm 0.009}$	$7.877_{\pm 0.009}$	$0.754_{\pm 0.001}$	$1.201_{\pm 0.009}$	$1.455_{\pm 0.012}$
**EntangledSBM w/o Velocity Conditioning**	$0.449_{\pm 0.000}$	$1.506_{\pm 0.005}$	$1.544_{\pm 0.005}$	$0.476_{\pm 0.000}$	$2.116_{\pm 0.005}$	$2.197_{\pm 0.005}$	$0.480_{\pm 0.000}$	$0.505_{\pm 0.004}$	$0.627_{\pm 0.005}$
**EntangledSBM w/ LV**	$0.308_{\pm 0.000}$	$0.175_{\pm 0.002}$	$0.256_{\pm 0.001}$	$0.302_{\pm 0.000}$	$0.340_{\pm 0.001}$	$0.565_{\pm 0.000}$	$0.312_{\pm 0.000}$	$0.198_{\pm 0.001}$	$0.321_{\pm 0.001}$
**EntangledSBM w/ CE**	$0.428_{\pm 0.000}$	$0.392_{\pm 0.005}$	$0.434_{\pm 0.006}$	$0.409_{\pm 0.000}$	$0.453_{\pm 0.008}$	$0.561_{\pm 0.009}$	$0.451_{\pm 0.000}$	$0.394_{\pm 0.003}$	$0.469_{\pm 0.004}$

G ALGORITHMS

Here, we provide the pseudocode for training (Alg 2) and inference (Alg 3) with EntangledSBM.

Algorithm 2 EntangledSBM **Training**

Input: Parameterized networks $\alpha_\theta(\boldsymbol{R}_t, \boldsymbol{V}_t) : \mathbb{R}^{n\times d} \times \mathbb{R}^{n\times d} \to \mathbb{R}^n$ and $\boldsymbol{h}_\theta(\boldsymbol{R}_t, \boldsymbol{V}_t) : \mathbb{R}^{n\times d} \times \mathbb{R}^{n\times d} \to \mathbb{R}^{n\times d}$, potential energy function $U(\boldsymbol{R}_t) : \mathbb{R}^{n\times d} \to \mathbb{R}$, distribution of target states $\pi_\mathcal{B}$, buffer size $|\mathcal{R}|$, batch size N_{batch}, number of samples M, number of rollouts N_{rollouts}, training steps per rollout N_{steps}, number of timesteps T

$\Delta t \leftarrow \frac{1}{T}$

$\mathcal{R} \leftarrow \{\}$ ▷ *Initialize empty replay buffer*

for rollout in $1, \dots, N_{\text{rollouts}}$ **do**

  for t in $1, \dots, T$ **do**

    Predict $\alpha^i_\theta(\boldsymbol{R}_t, \boldsymbol{V}_t) \in \mathbb{R}$ and $\boldsymbol{h}^i_\theta(\boldsymbol{R}_t, \boldsymbol{V}_t) \in \mathbb{R}^d$ with parameterized neural network where $\alpha^i_\theta(\boldsymbol{R}_t, \boldsymbol{V}_t) \geq 0$ is enforced with `softplus` activation

    $\boldsymbol{s}_i \leftarrow \nabla_{\boldsymbol{r}^i_t} \log \pi_\mathcal{B}$, $\hat{\boldsymbol{s}}_i \leftarrow \boldsymbol{s}_i / \|\boldsymbol{s}_i\|$

    Compute bias force

$$\boldsymbol{b}^i_\theta(\boldsymbol{R}_t, \boldsymbol{V}_t) \leftarrow \alpha^i_\theta(\boldsymbol{R}_t, \boldsymbol{V}_t)\hat{\boldsymbol{s}}_i + \left(\boldsymbol{I} - \hat{\boldsymbol{s}}_i\hat{\boldsymbol{s}}_i^\top\right) \boldsymbol{h}^i_\theta(\boldsymbol{R}_t, \boldsymbol{V}_t)$$

    Generate M discrete trajectories $\{\boldsymbol{X}_{0:T}\}_{j=1}^M$ with current bias $\boldsymbol{b}^i_\theta$ following (6) with Euler-Maruyama integration for each particle i

$$\boldsymbol{r}^i_{t+1} = \boldsymbol{r}^i_t + \boldsymbol{v}^i_t(\boldsymbol{R}_t)\Delta t + \boldsymbol{\Sigma}\boldsymbol{b}^i_\theta(\boldsymbol{R}_t, \boldsymbol{V}_t)\Delta t + \boldsymbol{\Sigma}\boldsymbol{\epsilon}_t$$

  end for

  $\mathcal{B} \leftarrow \mathcal{B} \cup \{\boldsymbol{X}_{0:T}\}_{j=1}^M$ ▷ *update buffer with discrete trajectories*

  for step in $1, \dots, N_{\text{steps}}$ **do**

    Sample batch $\{\boldsymbol{X}_{0:T}\}_{j=1}^{N_{\text{batch}}}$ from buffer $\mathcal{R}$

    Compute cross-entropy objective $\mathcal{L}_{\text{CE}}$ with (13)

    Update θ with $\nabla_\theta \mathcal{L}_{\text{CE}}$

  end for

end for

return parameterized $\alpha^i_\theta(\boldsymbol{R}_t, \boldsymbol{V}_t)$ and $\boldsymbol{h}^i_\theta(\boldsymbol{R}_t, \boldsymbol{V}_t)$

Algorithm 3 EntangledSBM **Inference**

Input: Trained networks $\alpha^i_\theta(\boldsymbol{R}_t, \boldsymbol{V}_t)$ and $\boldsymbol{h}^i_\theta(\boldsymbol{R}_t, \boldsymbol{V}_t)$, potential energy function $U(\boldsymbol{R}_t)$, initial state $\boldsymbol{X}_0 = (\boldsymbol{R}_0, \boldsymbol{V}_0)$, target state $\boldsymbol{X}_T = (\boldsymbol{R}_T, \boldsymbol{V}_T)$, time steps T, friction γ

$\Delta t \leftarrow \frac{1}{T}$, $\boldsymbol{R}_t \leftarrow \boldsymbol{R}_0$, $\boldsymbol{V}_t \leftarrow \boldsymbol{V}_0$

$\mathcal{P} \leftarrow \{\}$ ▷ *initialize path*

for t in $0, \dots, T$ **do**

  Predict $\alpha^i_\theta(\boldsymbol{R}_t, \boldsymbol{V}_t)$ and $\boldsymbol{h}^i_\theta(\boldsymbol{R}_t, \boldsymbol{V}_t)$ with parameterized neural network where $\alpha^i_\theta(\boldsymbol{R}_t, \boldsymbol{V}_t) \geq 0$ is enforced with `softplus` activation

  $\boldsymbol{s}_i \leftarrow \nabla_{\boldsymbol{r}^i_t} \log \pi_\mathcal{B}$, $\hat{\boldsymbol{s}}_i \leftarrow \boldsymbol{s}_i / \|\boldsymbol{s}_i\|$

  Compute bias force

$$\boldsymbol{b}^i_\theta(\boldsymbol{R}_t, \boldsymbol{V}_t) \leftarrow \alpha^i_\theta(\boldsymbol{R}_t, \boldsymbol{V}_t)\hat{\boldsymbol{s}}_i + \left(\boldsymbol{I} - \hat{\boldsymbol{s}}_i\hat{\boldsymbol{s}}_i^\top\right) \boldsymbol{h}^i_\theta(\boldsymbol{R}_t, \boldsymbol{V}_t)$$

  Generate M discrete trajectories $\{\boldsymbol{X}_{0:T}\}_{j=1}^M$ with current bias $\boldsymbol{b}^i_\theta$ following (6) with Euler-Maruyama integration for each particle i

$$\boldsymbol{r}^i_{t+1} = \boldsymbol{r}^i_t + \boldsymbol{v}^i_t(\boldsymbol{R}_t)\Delta t + \boldsymbol{\Sigma}\boldsymbol{b}^i_\theta(\boldsymbol{R}_t, \boldsymbol{V}_t)\Delta t + \boldsymbol{\Sigma}\boldsymbol{\epsilon}_t$$

  Append to path $\mathcal{P} \leftarrow \mathcal{P} \cup \{\boldsymbol{R}_t\}$

end for

return path $\mathcal{P}$
